# Metformin accelerates zebrafish heart regeneration by inducing autophagy

**DOI:** 10.1038/s41536-021-00172-w

**Published:** 2021-10-08

**Authors:** Fangjing Xie, Shisan Xu, Yingying Lu, Kin Fung Wong, Lei Sun, Kazi Md Mahmudul Hasan, Alvin C. H. Ma, Gary Tse, Sinai H. C. Manno, Li Tian, Jianbo Yue, Shuk Han Cheng

**Affiliations:** 1 Department of Biomedical Sciences, City University of Hong Kong, Hong Kong, China; 2 School of Life Sciences, Guangxi Normal University, Guilin, China; 3 Guangxi Universities Key Laboratory of Stem Cell and Biopharmaceutical Technology, Guangxi Normal University, Guilin, China; 4 City University of Hong Kong Shenzhen Research Institute, Shenzhen, China; 5 Department of Biomedical Engineering, The Hong Kong Polytechnic University, Hong Kong, China; 6 Department of Health Technology and Informatics, The Hong Kong Polytechnic University, Hong Kong, China; 7 Kent and Medway Medical School, Canterbury, UK

**Keywords:** Macroautophagy, Heart failure

## Abstract

Metformin is one of the most widely used drugs for type 2 diabetes and it also exhibits cardiovascular protective activity. However, the underlying mechanism of its action is not well understood. Here, we used an adult zebrafish model of heart cryoinjury, which mimics myocardial infarction in humans, and demonstrated that autophagy was significantly induced in the injured area. Through a systematic evaluation of the multiple cell types related to cardiac regeneration, we found that metformin enhanced the autophagic flux and improved epicardial, endocardial and vascular endothelial regeneration, accelerated transient collagen deposition and resolution, and induced cardiomyocyte proliferation. Whereas, when the autophagic flux was blocked, then all these processes were delayed. We also showed that metformin transiently enhanced the systolic function of the heart. Taken together, our results indicate that autophagy is positively involved in the metformin-induced acceleration of heart regeneration in zebrafish and suggest that this well-known diabetic drug has clinical value for the prevention and amelioration of myocardial infarction.

## Introduction

Autophagy is an evolutionarily conserved cellular catabolic degradation process, which occurs ubiquitously in all eukaryotic cells^1^. Autophagy maintains homeostasis in the heart by removing misfolded proteins and damaged organelles such as the mitochondria and endoplasmic reticulum, and it also protects cardiomyocytes against diverse pathological stressors^2,3^. During myocardial ischemia, the activation of autophagy by Ras homology enriched in brain (Rheb), adenosine monophosphate-activated protein kinase (AMPK), and glycogen synthase kinase-3β (GSK-3β), reduces damage to the myocardium by supplying energy, eliminating damaged mitochondria, and decreasing oxidative stress^4–6^. Conditional knockout of the cardiac-specific *Dynamin-related protein 1* (*Drp1*) gene in mice leads to increased cardiomyocyte death due to defective mitophagy, which results in the accumulation of damaged mitochondria during reperfusion^7^. Autophagy-related 5 protein (Atg5), a key component of one ubiquitin-like protein conjugation system (i.e., the Atg12 conjugation system), is essential for autophagy^8^. Mice with tamoxifen-inducible cardiac-specific *atg5* deletion show cardiac hypertrophy, left ventricular dilatation, and contractile dysfunction, with significant accumulation of damaged mitochondria and disorganized sarcomeres^9^. Moreover, *Atg5*-deficient mice die within 1 day of birth with reduced amino acid concentrations in the plasma^10^. Autophagy is also beneficial for cardiac remodeling after myocardial infarction (MI). For example, following MI in mice, Parkin plays a critical role in the elimination of dysfunctional mitochondria via autophagy, whereas Parkin deficiency impairs mitophagy and exacerbates cardiac dysfunction and dilation^11,12^. In addition, mammalian STE20-like protein kinase 1 (Mst1; a serine/threonine kinase that can be triggered by oxidative stress), negatively regulates autophagy via the phosphorylation of Beclin1 at threonine 108, and this phosphorylation event enhances the interaction between Beclin1 and Bcl-2 to activate Bax and stimulate apoptosis^13^. Knockout of the *Mst1* gene reduces the size of the infarct and improves cardiac function in mice subjected to MI by stimulating autophagy and blocking apoptosis^13^.

Metformin (N, N-dimethylbiguanide) is a first-line drug for treating type 2 diabetes (T2D). However, in addition to its anti-T2D effects, accumulating data indicate that it also has a cardiovascular protective effect and potentially reduces the occurrence of MI, heart failure, diabetic cardiomyopathy, and cardiac hypertrophy^14^. Although the exact molecular mechanisms of the therapeutic action of metformin are not yet fully understood, at least three major molecular targets of the drug have been identified. These include complex I of the mitochondrial electron transport chain (ETC), AMPK, and the mechanistic target of rapamycin complex 1 (mTORC1)^15^. Activation of AMPK can directly phosphorylate autophagy-related proteins, including ULK1^16,17^, Atg9^18^, Vps34^19^, Beclin1^20^, and Atg14L^21,22^, thereby enhancing autophagy. Moreover, the inhibition of mTORC1 activates ULK1^17^ and transcription factor EB (TFEB)^23,24^, which initiates autophagy.

MI is an acute cardiac injury caused by blockage of the coronary blood supply, usually due to the rupture of an atherosclerotic plaque^25^. Studies have shown that in humans and adult mice, cardiomyocytes can regenerate, but the regenerative capacity is very low, i.e., < 1% annually^26,27^. This is, therefore, a very ineffective way to replace the diseased tissue. In contrast to mammals, zebrafish Danio rerio possess a highly efficient adult heart regeneration capacity, and so they are a valuable model organism for studying the regenerative response following myocardial damage due to resection of the ventricular apex^28^, or cryoinjury^29–31^, or genetic cardiomyocyte depletion^32^. In zebrafish, heart regeneration is a dynamic process, and diverse signals and factors are recruited to clear damaged or necrotic tissue; regulate inflammation; deposit and resolve transient fibrosis; and stimulate myocardial, epicardial, and endocardial regeneration and neovascularization^25^. Indeed, the mechanisms that are involved in regeneration have gradually been deciphered in this model. However, few pharmacological or genetic strategies have been reported that stimulate or accelerate zebrafish cardiomyocyte proliferation and heart regeneration following injury. Given that metformin can induce autophagy, it is of great interest to study whether this drug might enhance the autophagic flux to improve zebrafish heart regeneration.

## Results

### Autophagy is upregulated during zebrafish heart regeneration

**Fig. 1:**
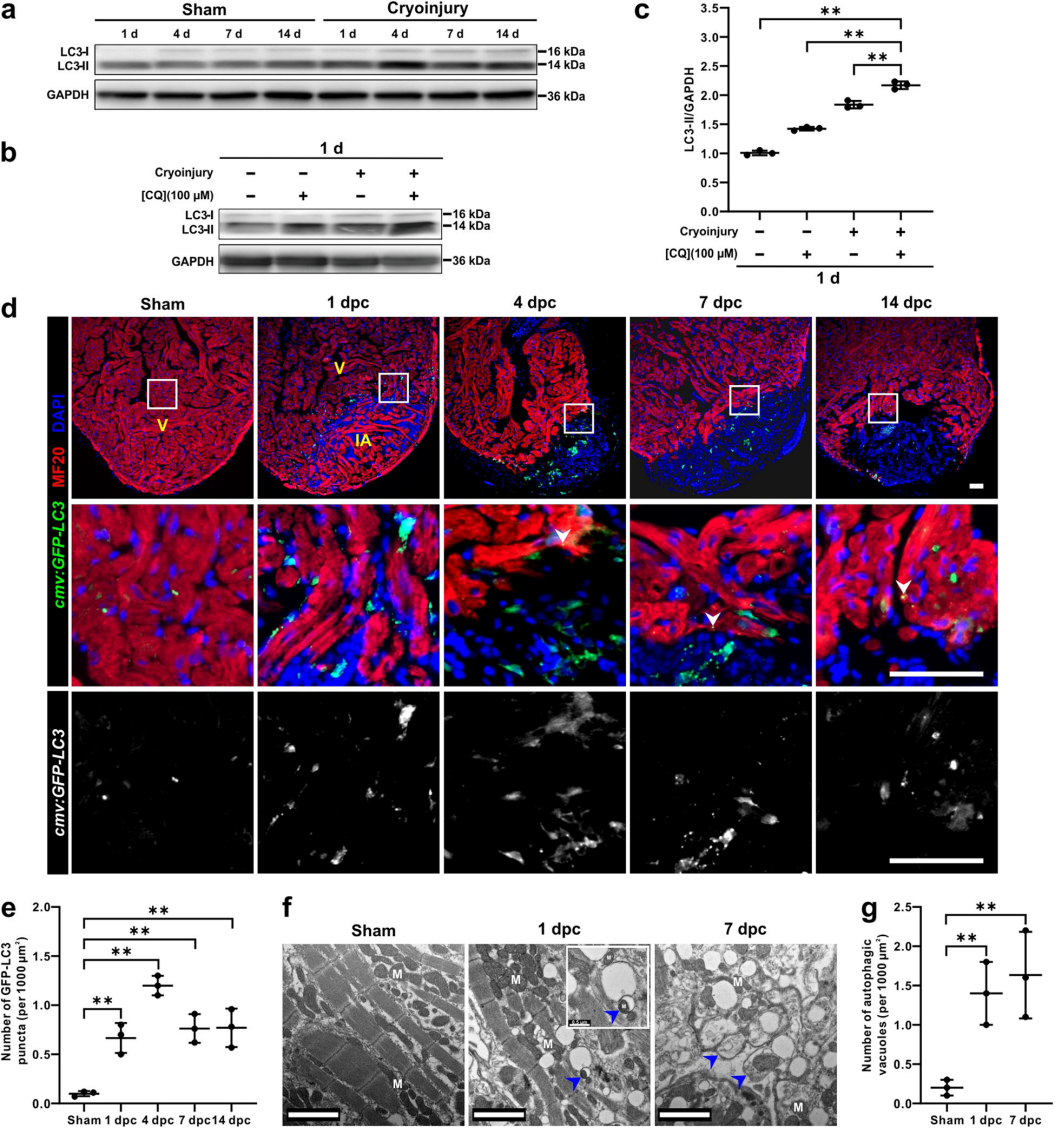
Autophagy is upregulated during zebrafish heart regeneration. **a** Western blot analysis was performed to detect the expression of LC3-I and LC3-II in intact (sham control) and regenerating hearts from 1 to 14 days. The protein from three hearts was loaded into each lane, and GAPDH was used as the loading control. **b**, **c** Autophagic flux assay with a western blot of LC3 was conducted (**b**). The expression of LC3-II was quantified. The data are presented as mean ± SD, *n* = 3 repeats, **P < 0.01 *vs* cryoinjured hearts with CQ treatment (**c**). **d**, **e** The sham (control) or cryoinjured hearts of *Tg*(*cmv*:*GFP-LC3*) fish were isolated, embedded in paraffin, sectioned, and dual-immunostained with an anti-GFP antibody to detect LC3 (in green) and an anti-MF20 antibody to detect the cardiomyocytes (in red), after which they were co-stained with DAPI to label the nuclei (in blue). IA: injured area; V: ventricle. The arrowheads show the GFP-LC3 puncta in cardiomyocytes. Scale bars: 50 µm (**d**). The number of GFP-LC3 puncta in sham and in the injured area at 1 dpc, 4 dpc, 7 dpc, and 14 dpc was quantified. The data are presented as mean ± SD, *n* = 3 hearts, **P < 0.01 *vs* sham (**e**). **f**, **g** Electron micrographs of an intact (sham) heart, and a regenerating heart in the IA at 1 dpc and 7 dpc. In the injured area, there was an accumulation of autophagic vacuoles (see blue arrowheads). M: mitochondria. Scale bars: 2 µm (**f**). The number of autophagic vacuoles in sham and the injured area at 1 dpc and 7 dpc was quantified. The data are presented as mean ± SD, *n* = 3 hearts, **P < 0.01 *vs* sham (**g**).

In the zebrafish heart following injury, significant changes in the morphology of the injured region indicate that regeneration might trigger certain mechanisms of cytoplasm remodelling^29^. To determine if autophagy participates in this process, we cryoinjured the hearts of adult zebrafish and assessed the expression of LC3 at different time points. LC3 in zebrafish is the autophagic protein orthologue of Atg8 in yeast and MAP1LC3 in mammals^33^. LC3-I, the phosphatidylethanolamine (PE) unconjugated form of LC3, is localized in the cytosol, whereas LC3-II, the PE-conjugated form of LC3, is most abundant in autophagosomal membranes. This latter is well established as a marker to monitor autophagosomal and autophagic activity^1^. We showed that the level of LC3-II was markedly increased from 1 day post cryoinjury (dpc) to 14 dpc when compared with uninjured hearts (Fig. 1a). Moreover, when cryoinjured fish were treated with chloroquine (CQ), which blocks the fusion of autophagosomes and lysosomes^34,35^, the level of LC3-II was further increased when compared with untreated cryoinjured fish or CQ-treated uninjured fish (Fig. 1b, c). In the *Tg*(*cmv*:*GFP-LC3*) line of fish, GFP-LC3 puncta were distinguished from background when they occupied more than one pixel of fluorescence signals as previously described^36^. The total number of GFP-LC3 puncta in the injured area was counted. We showed that the number of GFP-LC3 puncta was low in the sham (control) hearts; however, they were significantly induced in the injured area from 1 to 14 dpc, and they peaked at 4 dpc (Fig. 1d, e). GFP-LC3 puncta were also detected in cardiomyocytes abutting the injured area at 4 dpc, 7 pdc, and 14 dpc (see white arrowheads in the middle panels of Fig. 1d). To investigate the morphological changes that occur in the heart following cryoinjury in further detail, we performed transmission electron microscopy (TEM). The TEM images showed an increase in the number of autophagic vacuoles in the injured area of the ventricle at 1 dpc and 7 dpc, when compared with the sham hearts (Fig. 1f, g). Damaged mitochondria were also observed to be eliminated by mitophagy (Fig. 1f). Taken together, these results suggest that autophagy is upregulated in the zebrafish heart following cryoinjury, which is consistent with the model of ventricular apex resection^37^.

### Metformin enhances autophagy and accelerates heart regeneration

**Fig. 2:**
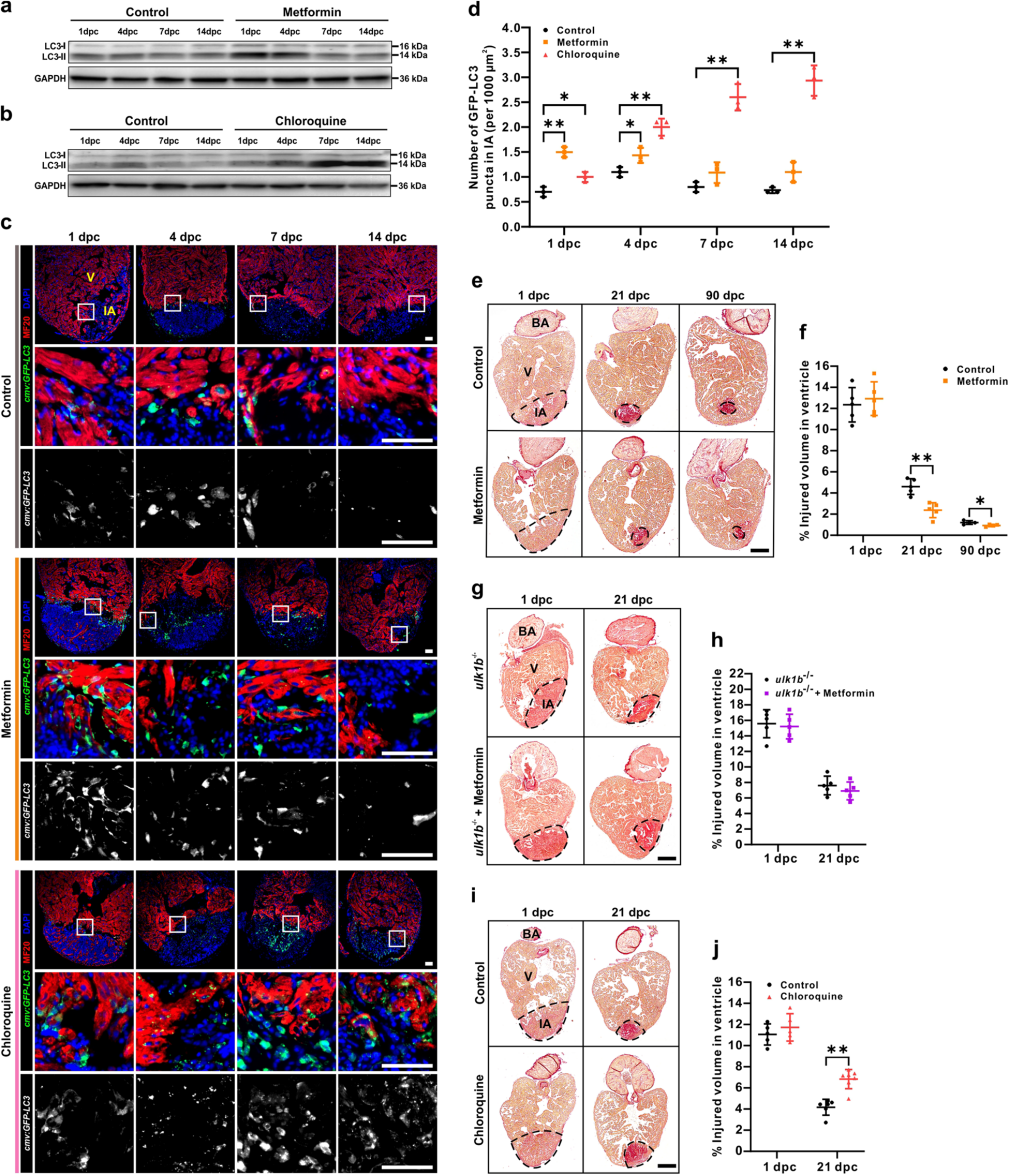
Metformin enhances the autophagic flux and accelerates heart regeneration. **a**, **b** Western blot analysis was performed in cryoinjured hearts treated with or without 50 µM metformin (**a**) or 100 µM CQ (**b**) to detect the expression of LC3-I and LC3-II from 1 to 14 dpc. The protein from three hearts was loaded into each lane, and GAPDH was used as the loading control. **c**, **d** After cryoinjury, the heart of *Tg*(*cmv*:*GFP-LC3*) fish treated with or without 50 µM metformin or 100 µM CQ, were isolated, fixed, sectioned and dual-immunostained with anti-GFP (green) and anti-MF20 (red), after which they were stained with DAPI (blue). IA: injured area; V: ventricle. Scale bars: 50 µm (**c**). The number of GFP-LC3 puncta in the injured area at 1 dpc, 4 dpc, 7 dpc, and 14 dpc was quantified. The data are presented as mean ± SD, *n* = 3 hearts, *P < 0.05, **P < 0.01 *vs* control (**d**). **e–j** Following cryoinjury, the AB fish were either untreated (control) or else they were treated with 50 µM metformin for up to 90 days (**e**, **f**), or they were treated with 100 µM CQ for up to 21 days (**i**, **j**); the *ulk1b* mutant fish generated by TALEN were also either untreated (control) or else they were treated with 50 µM metformin for up to 21 days (**g**, **h**). Paraffin sections were then prepared and stained with Picro Sirius red. BA: bulbous arteriosus. Scale bars: 200 µm. The injured volume percentage was then quantified in the ventricle of the control and metformin (**f**, **h**) or CQ (**j**) treatment groups at the different time points. The data are presented as mean ± SD, *n* = 4 to 7 hearts, *P < 0.05, **P < 0.01 *vs* control.

To examine if metformin might have an effect on the autophagic flux, zebrafish treated with or without metformin (50 µM) were cryoinjured, and the expression of LC3 in the hearts was then evaluated. We showed that the amount of LC3-II was markedly increased at 1 dpc and 4 dpc in fish treated with metformin, when compared with the controls (Fig. 2a). Consistently, immunostaining analysis also showed that GFP-LC3 puncta were significantly increased in injured hearts in metformin-treated fish at 1 dpc and 4 dpc, when compared with the controls (Fig. 2c, d). To investigate the effect of metformin on heart regeneration, one group of ventricle-cryoinjured zebrafish was cultured in fish water containing 50 µM metformin, and this was replaced every day for 90 days, whereas another group was maintained in fish water alone (control) for the same period. Hearts were isolated and fixed at 1 dpc, 21 dpc, and 90 dpc, after which a series of sections were prepared and stained with Picro Sirius red, and the volume of the injured area was then calculated. At 1 dpc, the percentage of the ventricle that consisted of the injured area in the control group was ~ 12.35% and that of the metformin treatment group was ~ 12.93%. Thus, the volume of the injured area was not significantly different between the two groups (*p* = 0.58; Fig. 2e, f). However, after 21 days, the percentage of the injured area of the control group was ~ 4.61%, whereas that in the metformin group was ~ 2.38%. Thus, the volume of the injured area in the metformin group was significantly smaller than that of the control group (*p* < 0.01; Fig. 2e, f). Finally, after 90 days, the percentage of the injured volume in the ventricle in the control and metformin treatment groups, was ~ 1.21% and ~ 0.93%, respectively. Thus, the volume of the injured area in the metformin group was still significantly smaller than that of the control group (*p* < 0.05; Fig. 2e, f). However, in an autophagy-deficient line, which was generated by transcription activator-like effector nuclease (TALEN)-mediated gene targeting of *ulk1b*^38^, a vital autophagy-related orthologue of human *ULK1*, metformin treatment failed to affect the volume of the injured area in the ventricle at 21 dpc, when compared to the control *ulk1b* mutant group (*p* = 0.38; Fig. 2g, h).

Torin 1 is a highly potent and selective inhibitor of mTOR^39,40^, which is known to promote autophagy^17,23,24^. We showed that when fish were treated with Torin 1 (25 nM), the level of phosphorylated mTOR decreased and the level of LC3-II increased from 1 to 7 dpc (Supplementary Fig. 1a). Similarly, Torin 1 significantly promoted autophagy in injured hearts from 1 to 7 dpc when compared with the solvent control (Supplementary Fig. 1b, c). The volume of the injured area in the Torin 1-treated group was also significantly smaller than that of the control group at 21 dpc (*p* < 0.01; Supplementary Fig. 1d, e). In summary, these results suggest that metformin or Torin 1 might promote heart regeneration by enhancing autophagy.

To further confirm the role of autophagy in heart regeneration, zebrafish hearts were cryoinjured and then the fish were treated with or without CQ (100 µM). We showed that LC3-II accumulated from 1 to 14 dpc in the hearts of CQ-treated fish when compared with the controls (Fig. 2b). Likewise, the number of GFP-LC3 puncta was also significantly higher in injured hearts treated with CQ from 1 to 14 dpc, when compared with the controls (Fig. 2c, d). Furthermore, after 21 days, the volume of the injured area in the CQ group was significantly larger than that of the control group (*p* < 0.01; Fig. 2i, j). These results clearly show that when autophagy was blocked by CQ treatment, then heart regeneration was delayed.

We also used high resolution, real-time echocardiography to non-invasively evaluate the changes of myocardial function during zebrafish heart regeneration (Supplementary Fig. 2a and Supplementary Videos 1–10). Longitudinal ventricular measurements by B-Mode imaging documented a remarkable difference in the systolic function in metformin-treated fish when compared with controls, as indicated by the fractional shortening (FS) and ejection fraction (EF) (Supplementary Fig. 2b, c). At 1 dpc and 28 dpc, FS in the control group was ~ 12.7% and ~ 19.9%, whereas that in the metformin treatment group was ~ 12.6% and ~ 20.0%. Thus, the FS was not significantly different between the two groups (Supplementary Fig. 2b). However, at 7 dpc, 14 dpc, and 21 dpc, the FS in the control group was ~ 17.0%, ~ 17.2% and ~ 21.7%, and that in the metformin treatment group was ~ 22.4%, ~ 22.1% and ~ 28.2%, respectively. Thus, at these time points, the FS in the metformin group was significantly higher than in the control group (*p* < 0.01; Supplementary Fig. 2b). The EF followed a similar pattern as the FS in that at 1 dpc and 28 dpc, there were no significant differences between the metformin and control groups, with values of ~ 24.5% and ~ 24.7%, respectively at 1 dpc and values of ~ 41.2% and ~ 42.3%, respectively at 28 dpc (Supplementary Fig. 2c). However, from 7 to 21 dpc, the EF in the metformin group was significantly higher than that of the control group with values of ~ 41.8% and ~ 30.8%, respectively, at 7 dpc (*p* < 0.01); ~ 40.0% and ~ 33.8%, respectively, at 14 dpc (*p* < 0.05); and ~ 50.3% and ~ 40.8%, respectively, at 21 dpc (*p* < 0.05) (Supplementary Fig. 2c). These results indicate that metformin treatment enhances the systolic function of the heart during regeneration.

### Metformin accelerates myocardium regeneration

Since we showed that metformin treatment accelerates heart regeneration after cryoinjury (Fig. 2, Supplementary Figs. 1 and 2), we next assessed if the metformin**-**enhanced autophagic flux might increase the total amount of cell proliferation. Paraffin sections of cryoinjured hearts were immunostained with the anti-PCNA antibody to label the nuclei of proliferating cells, and then they were counterstained with DAPI to label the nuclei of all the cells (Supplementary Fig. 3**)**. We found that compared with untreated controls, metformin significantly induced the proliferation of cells in the injured area at 4 dpc and 7 dpc. In contrast, the number of proliferating cells was significantly reduced at 7 dpc following treatment with CQ (Supplementary Fig. 3). We also examined whether activation or inhibition of autophagy might affect apoptosis in the injured area after cryoinjury (Supplementary Fig. 4). The TUNEL assay showed that in control, metformin-, or CQ-treated hearts, the numbers of apoptotic cells were high at 1 dpc and were considerably decreased by 7 dpc, but no significant differences were observed among these groups (Supplementary Fig. 4).

**Fig. 3:**
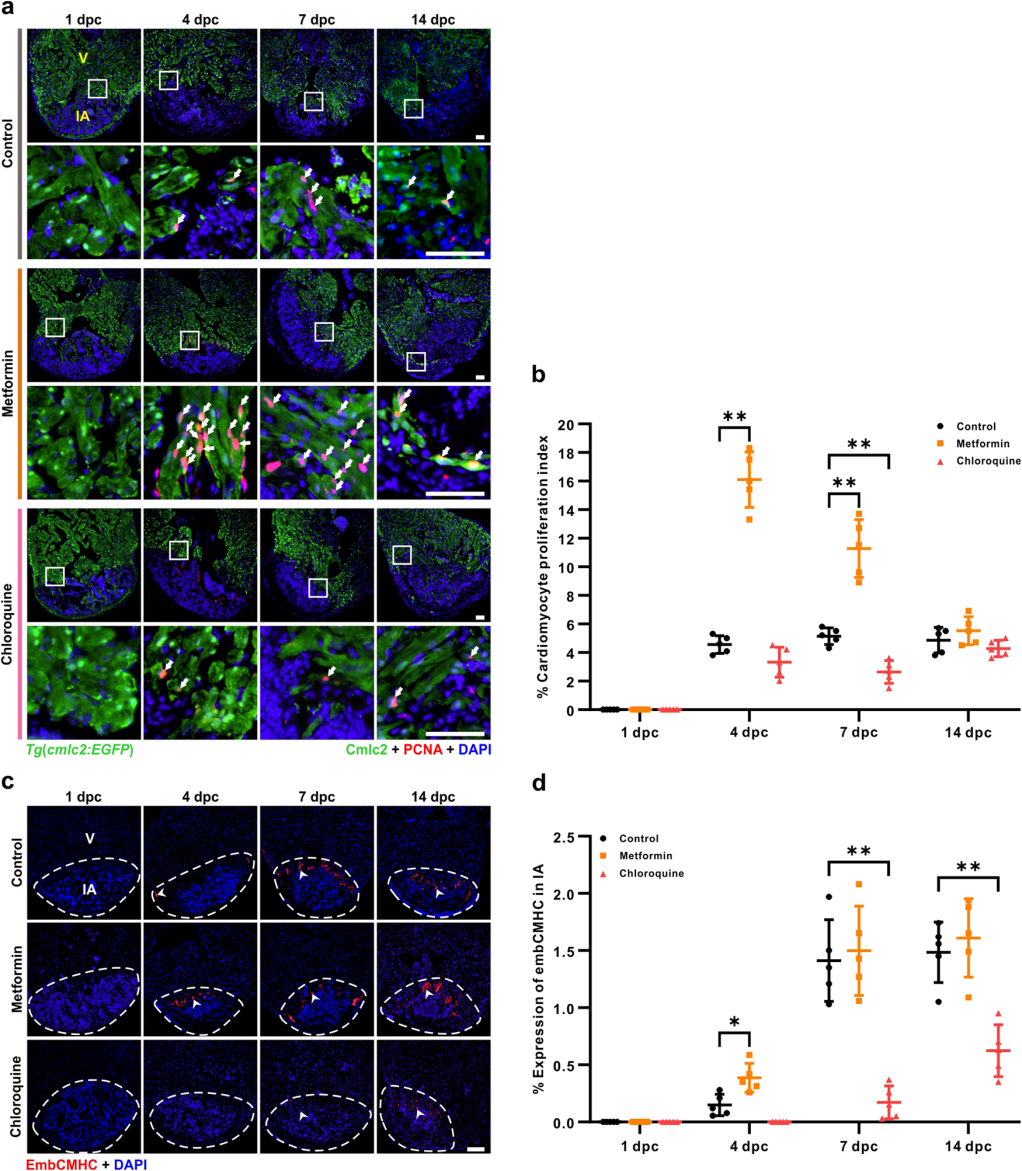
Metformin accelerates regeneration of the myocardium. **a, b** After cryoinjury, the *Tg*(*cmlc2*:*EGFP*) fish were maintained in untreated fish water (control) or in fish water containing 50 µM metformin or 100 µM CQ for the indicated days. The hearts were then isolated, fixed, sectioned and dual-immunostained with an anti-GFP antibody to identify the cardiomyocytes (in green) and an anti-PCNA antibody to identify proliferating cells (in red), after which they were co-stained with DAPI to label the nuclei (in blue). V: ventricle; IA: injured area. The white arrows show the proliferating cardiomyocytes. Scale bars: 50 µm (**a**). The proliferating cardiomyocytes in the untreated control, metformin and CQ treated fish at 1 dpc, 4 dpc, 7 dpc, and 14 dpc were quantified. The data are presented as mean ± SD, *n* = 5 hearts, **P < 0.01 *vs* control (**b**). **c**, **d** After heart cryoinjury, AB fish were maintained in untreated fish water (control) or in fish water containing 50 µM metformin or 100 µM CQ. The hearts were prepared, and immunostained with an anti-embCMHC (N2.261) antibody for the identification of embryonic cardiac myosin heavy chain (in red) and then they were labeled with DAPI. The arrowheads show the signal of embCMHC. Scale bar: 100 µm (**c**). The per**c**entage of embCMHC positive cells in the control, metformin and CQ treated fish at 1 dpc, 4 dpc, 7 dpc, and 14 dpc was quantified. The data are presented as mean ± SD, n = 5 hearts, *P < 0.05, **P < 0.01 *vs* control (**d**).

We also evaluated the effect of metformin on the activation of myocardial cells during regeneration in the *Tg*(*cmlc2*:*EGFP*) line of zebrafish (Fig. 3). Paraffin sections were prepared and dual-immunostained with an anti-GFP antibody to identify the cardiomyocytes (in green) and an anti-PCNA antibody to label the nuclei of proliferating cells (in red) (Fig. 3a). The numbers of proliferating cardiomyocytes (see white arrows) were quantified, and these are presented as a percentage of the total number of cardiomyocytes in the wound border zone (within 100 µm from the injured area) (Fig. 3b**)**. Significant differences were observed between the metformin and control groups, such that in the metformin-treated samples, there were significantly greater numbers of proliferating cardiomyocytes than in the control at 4 dpc (i.e., ~16.1% compared to ~ 4.6%, respectively) and at 7 dpc (i.e., ~ 11.3% compared to ~ 5.1%, respectively) (Fig. 3a, b). However, at 1 dpc, no proliferating cardiomyocytes were detected in either the metformin or control groups, and at 14 dpc, the amount of cardiomyocyte proliferation in the metformin treatment group was similar to that in the control (i.e., ~ 5.5% compared to ~ 4.9%, respectively) (Fig. 3a, b).

Cell cycle re-entry not only results in the resumption of mitosis, but it can also lead to polyploidization and eventually binucleation^41,42^. In addition, it has been reported that during zebrafish heart regeneration, newly regenerated cardiomyocytes are derived from existing differentiated cardiomyocytes^32,43,44^. We showed that autophagy was activated in immature cardiomyocytes (see white arrowheads) at 4 dpc, 7 dpc, and 14 dpc (Supplementary Fig. 5). Next, we investigated if metformin might affect the proliferation of immature cardiomyocytes, and so cryoinjured ventricles were immunostained with the N2.261 monoclonal antibody to mark the embryonic cardiac myosin heavy chain (embCMHC) (Fig. 3c). This antibody was initially shown to detect neonatal muscle MHC in mammals^45,46^, but more recently it was also demonstrated to detect embryo-specific myosin in zebrafish^47,48^. Our results showed that more immature cardiomyocytes appeared in the injured area of metformin-treated fish when compared with control fish at 4 dpc (Fig. 3c, d). These results validate the effect of metformin on promoting regeneration of the myocardium. In contrast, the CQ-treated group exhibited significantly lower cardiomyocyte proliferation than the control group at 7 dpc (Fig. 3a, b). Furthermore, in the heart of CQ-treated fish, embCMHC was either low or not detected at all at 4 dpc, and it was only weakly detected at 7 dpc and 14 dpc (Fig. 3c, d). These data indicate that when the proliferation of cardiomyocytes and formation of immature cardiomyocytes are both inhibited with CQ, then this results in a delay in myocardium regeneration. In summary, these results suggest that autophagy is positively involved in the metformin-stimulating regeneration of the myocardium.

### Metformin accelerates regeneration of the endocardium and vascular endothelium

**Fig. 4:**
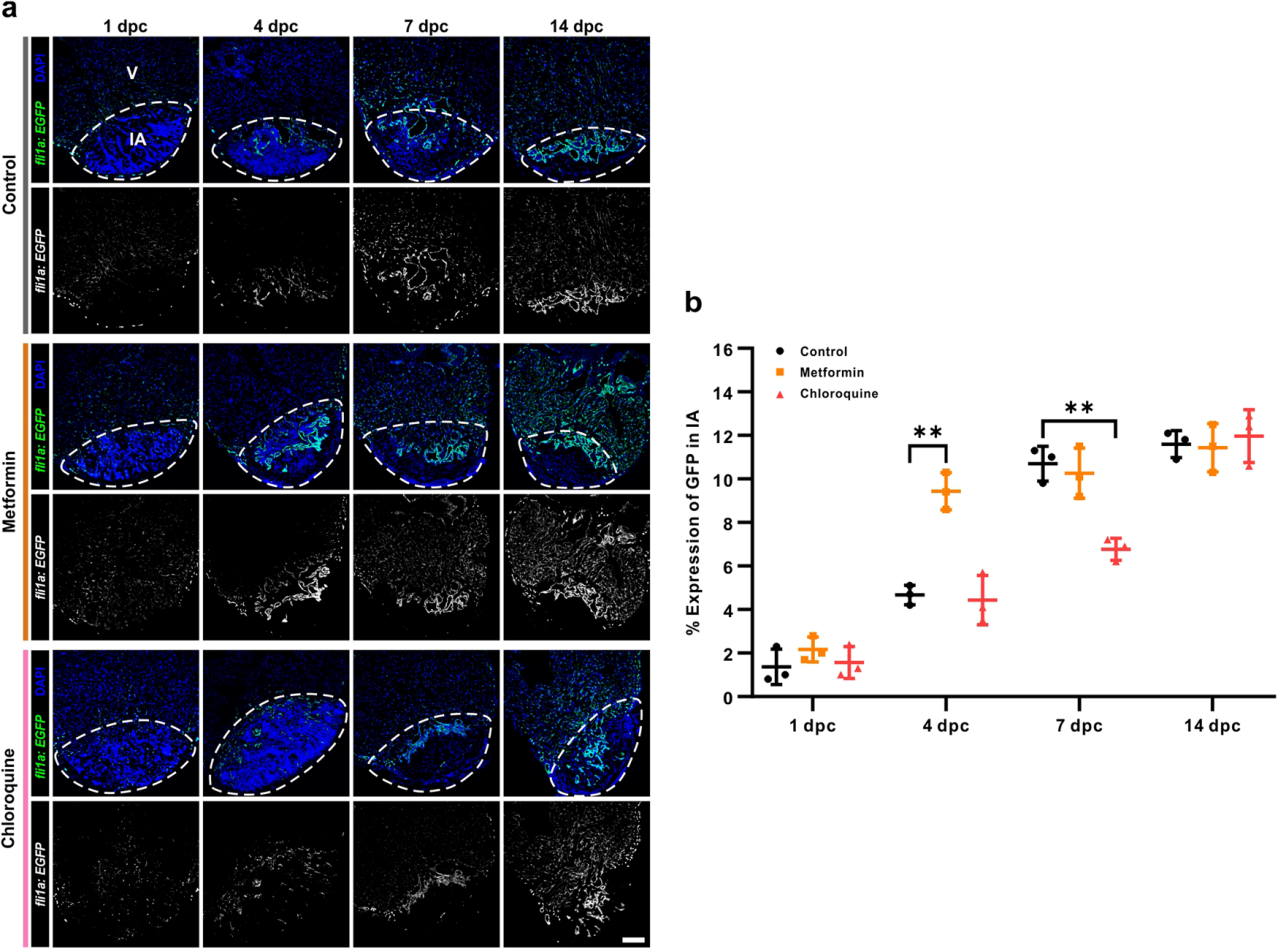
Metformin accelerates regeneration of the endocardium and vascular endothelium. **a** After heart cryoinjury, the *Tg*(*fli1a*:*EGFP*) fish, which express EGFP specifically in the endocardium and vascular endothelium, were maintained in untreated fish water (control) or in fish water containing 50 µM metformin or 100 µM CQ for 1–14 days. The hearts were then isolated, fixed, sectioned and immunostained with an anti-GFP antibody (in green), and co-labeled with DAPI to show the nuclei (in blue). V: ventricle; IA: injured area. Scale bar: 100 µm. **b** The percentage of Fli1a expressing cells in the injured area of the control, metformin and CQ treated fish at 1 dpc, 4 dpc, 7 dpc, and 14 dpc was quantified. The data are presented as mean ± SD, *n* = 3 hearts, **P < 0.01 *vs* control.

In addition to cardiomyocyte proliferation, it has been reported that endothelial and epicardial cells are activated after heart injury in order to provide the cells required for neovascularization and extracellular matrix (ECM) re-construction^49^. Thus, we investigated the effect of metformin on the activation of endocardial and vascular endothelial cells during regeneration in the *Tg*(*fli1a*: *EGFP*) line of zebrafish (Fig. 4), where EGFP is expressed specifically in the endocardial and vascular endothelial cells of the heart^30^. We showed that GFP fluorescence was detected in both the metformin-treated and control groups at 1 dpc, 4 dpc, 7 dpc, and 14 dpc, especially in the upper margin of the injured area adjacent to the uninjured tissue (Fig. 4a). This suggests that endothelial cells (and thus blood vessels) might regenerate in the injured area for a few days following cryoinjury. When the percentage of Fli1a*-*expressing cells in the injured area was calculated, significant differences were observed at 4 dpc (*p* < 0.01) when comparing the metformin-treated and control groups, but at 1 dpc, 7 dpc, and 14 dpc, no significant differences were found between these two groups (Fig. 4a, b). These data indicate that endothelial cells were activated after injury in both the control and metformin-treated groups, but regeneration of the endocardium and vascular endothelium was accelerated in the metformin-treated group. We also investigated the effect of CQ on the activation of endocardial and vascular endothelial cells during regeneration in *Tg*(*fli1a*: *EGFP*) fish. Our data showed that there was a significantly lower level of GFP in the CQ-treated fishes when compared with the control fishes at 7 dpc (*p* < 0.01) (Fig. 4a, b). Thus, these data indicate that CQ treatment appears to delay regeneration of the endocardium and vascular endothelium.

### Metformin accelerates epicardium regeneration

**Fig. 5:**
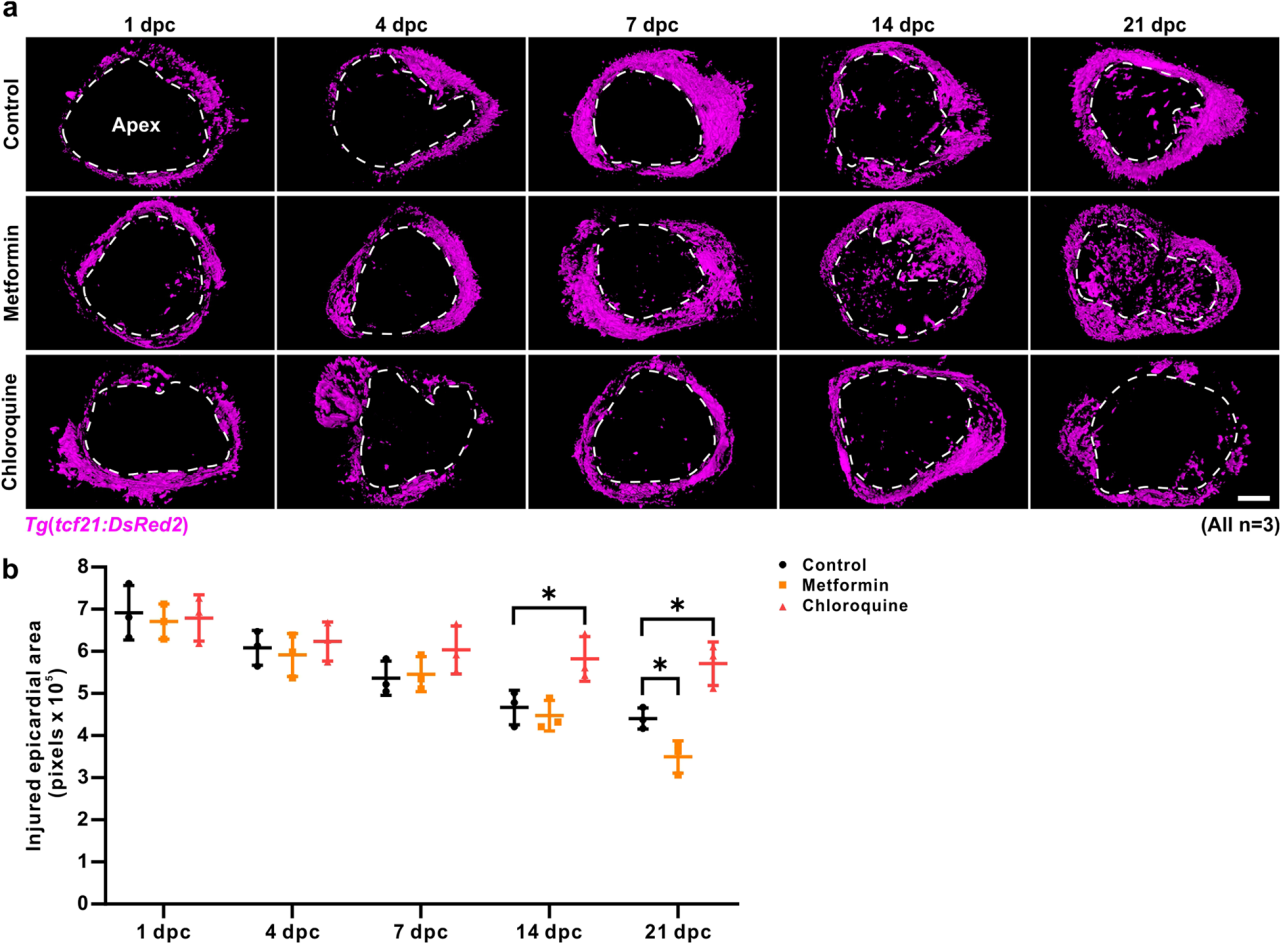
Metformin accelerates regeneration of the epicardium. **a** After cryoinjury, the *Tg*(*tcf21:DsRed2*) line of fish, which express DsRed2 (in pink) specifically in the epicardium were maintained in untreated fish water (control) or in fish water containing 50 µM metformin or 100 µM CQ for 1–21 days. The hearts were then isolated and directly embedded in capillaries with 1% low melting agarose gel, after which a Z-stack of frontal view images through the ventricular apex were acquired ex vivo using light sheet fluorescence microscopy. The Z-stack images were displayed in three dimensions with surface render mode. The dashed line in each image indicates the injured area. Scale bar: 200 µm. **b** The injured epicardial area of the control, metformin, and CQ-treated fish at 1 dpc, 4 dpc, 7 dpc, 14 dpc, and 21 dpc were quantified. The data are presented as mean ± SD, *n* = 3 hearts, *P < 0.05 *vs* control.

We also investigated the effect of metformin on the activation of epicardial cells during regeneration in the *Tg*(*tcf21:DsRed2*) zebrafish line (Fig. 5), where DsRed2 fluorescence is expressed specifically in the epicardium^50^. The hearts were cryoinjured, and then the fish were maintained in untreated fish water (control) or in fish water containing 50 µM metformin or 100 µM CQ. At 1 dpc, 4 dpc, 7 dpc, 14 dpc, and 21 dpc, the hearts were collected and directly subjected (i.e., ex vivo) to imaging with the light sheet fluorescence microscope. Z-stacks of optical sections through the frontal view of the ventricular apex were obtained, and these were projected as single images displayed in three dimensions using the surface render mode (Fig. 5a). In the cryoinjured hearts, DsRed2 fluorescence was detected in the untreated controls and in the metformin- and CQ-treated groups at 1 dpc, 4 dpc, 7 dpc, 14 dpc, and 21 dpc (Fig. 5a). In the metformin-treated group, at 21 dpc the expression of DsRed2 was more widespread throughout the surface of the injured area, when compared with the untreated controls (*p* < 0.05) (Fig. 5a, b). In contrast, in the CQ-treated group, the expression of DsRed2 was less widespread throughout the surface of the injured area when compared with the untreated controls at both 14 dpc and 21 dpc (Fig. 5a, b). These data indicate that epicardial cells are regenerated in the injured area over time, and that metformin and CQ appear to accelerate and delay epicardial regeneration, respectively.

### Metformin accelerates collagen deposition in the injured area

**Fig. 6:**
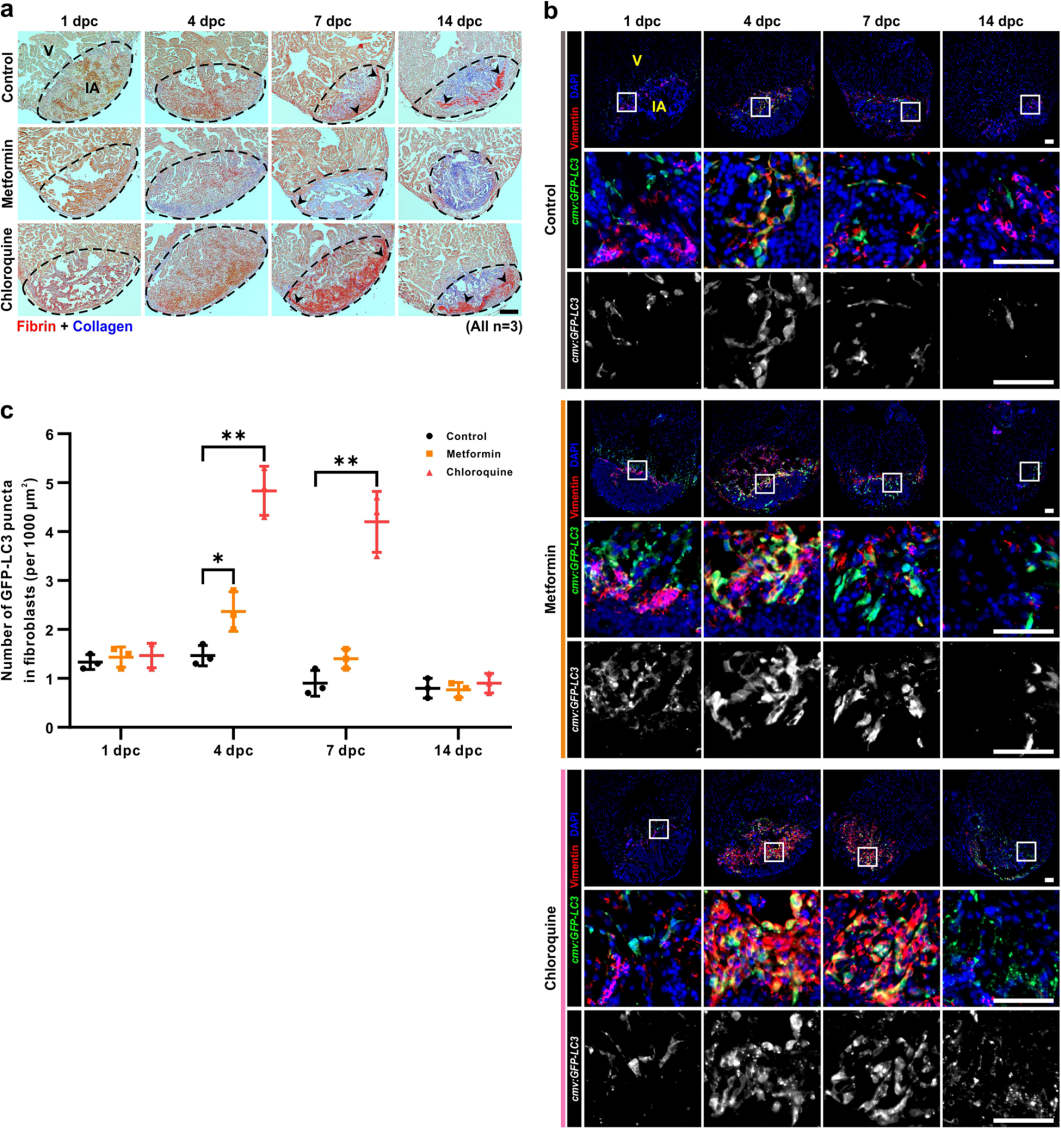
Metformin accelerates collagen deposition in the injured area. **a** The hearts of AB fish were cryoinjured, and then the fish were maintained in untreated fish water (control) or in fish water containing 50 µM metformin or 100 µM CQ for 1–14 days. Sections were then prepared and stained with the MSB trichrome stain to show fibrin (in dark red) and collagen (in blue). V: ventricle; IA: injured area. Scale bar: 100 µm. **b**, **c** The hearts of *Tg*(*cmv*:*GFP-LC3*) fish were cryoinjured, and then the fish were maintained in untreated fish water (control) or in fish water containing 50 µM metformin or 100 µM CQ for 1-14 days. The hearts were then isolated, fixed, sectioned, and dual-immunostained with an anti-GFP antibody to label LC3 (in green) and an anti-vimentin antibody to label fibroblasts (in red), after which they were labeled with DAPI to show the nuclei (in blue). Scale bars: 50 µm (**b**). The numbers of GFP-LC3 puncta in the fibroblasts in the injured area of the control, metformin and CQ treated fish were quantified. The data are presented as mean ± SD, *n* = 3 hearts, *P < 0.05, **P < 0.01 *vs* control (**c**).

In zebrafish, transient fibrosis is essential for cardiomyocyte proliferation and heart regeneration^51,52^. We, therefore, investigated if metformin might affect the deposition of collagen in the injured area following cryoinjury via the use of Martius, Scarlet, and Blue (MSB) staining to label the collagen (blue) and fibrin (dark red) (Fig. 6a). We showed that there was little collagen deposition in the injured area at 1 dpc in the metformin-treated and control groups (Fig. 6a). At 4 dpc, 7 dpc, and 14 dpc, relatively more collagen was observed in the injured area in the metformin-treated groups than in the controls; moreover, at 7 dpc and 14 dpc, less fibrin remained in the metformin-treated groups (Fig. 6a). In contrast, in the CQ-treated group, relatively little collagen was deposited in the injured area at 7 dpc with much of the injured area being taken up by fibrin (Fig. 6a).

Physiologically, fibroblasts are responsible for the homeostasis of components of the ECM, such as collagen, fibronectin and integrin^53^. Thus, we wanted to find out if metformin might modulate the expression of autophagy in fibroblasts following cryoinjury. Using the *Tg*(*cmv*:*GFP-LC3*) line of fish, we showed that with metformin treatment, at 1 dpc the level of autophagy was overall relatively high in the injured area, but it was low in fibroblasts (Fig. 6b, c). At 4 dpc, treatment with metformin apparently increased the number of vimentin-positive fibroblasts in the injured area and the activity of autophagy in fibroblasts, when compared with the control (Fig. 6b, c). However, at 7 dpc and 14 dpc, the levels of autophagy in fibroblasts were low in both the metformin-treated groups and the controls (Fig. 6b, c). Following treatment with CQ, far more vimentin-positive fibroblasts had accumulated in the injured area, and autophagy was blocked in fibroblasts at 4 dpc and 7 dpc (Fig. 6b, c).

## Discussion

In the zebrafish heart, cryoinjury is generally considered to more accurately represent the cellular events that occur during MI in humans, than does ventricular resection. This is because similar to MI, shortly after cryoinjury there is massive death of ventricular cardiomyocytes and inflammation, as well as a higher level of collagen deposited. In addition, during the 60–90 days following cryoinjury, the damaged tissue is progressively replaced by newly regenerated cardiomyocytes^29,30^. Autophagy plays a fundamental role in providing a source of energy for cell survival. It maintains cellular homeostasis by clearing cytoplasmic debris and defective organelles, and it is also essential for the development and differentiation of cells^54^. To investigate if autophagy is involved in heart regeneration in zebrafish, we cryoinjured the hearts and observed the induction of autophagy via immunoblotting, immunostaining, and TEM methodologies. We demonstrated that indeed autophagy is an endogenous pro-regenerative response in the adult zebrafish heart (Fig. 1). Autophagy was induced in the injured area during heart regeneration being activated in several cell types, including cardiomyocytes abutting the injured area; immature cardiomyocytes; and vimentin-positive fibroblasts (Figs. 1 and 6, Supplementary Fig. 5).

In the zebrafish heart, cells of the epicardium, endocardium, and vascular endothelium are activated on the first day after cryoinjury (Figs. 4 and 5). These non-muscle cells play an essential role in providing an environment that facilitates myocardial proliferation. Indeed, the epicardium is known to be a dynamic signaling center for cardiac repair, and epicardial cells undergo morphological changes as early as 12 h post amputation when they detach from their neighboring cells^30^. Epicardial cells are highly proliferative and they invade the underlying injured area and give rise to epicardial-derived cells (EPDCs), such as EPDC-derived cardiac fibroblasts, which secrete ECM proteins in response to other cell types survival, proliferation and migration^49,55^. Pre-existing endocardial and vascular endothelial cells act in a similar way to the epicardium following activation. Those that surround the injured area proliferate robustly from 3 to 5 dpc, and they contribute to the overall expansion of these cell types until 7 dpc^56^. Furthermore, revascularization is initiated as early as 15 h after injury, with vascular sprouting observed in the injured area. Indeed, blocking this early revascularization process, inhibits the regenerative capacity of the injured heart^57^.

Through the activation of AMPK and inhibition of mTORC1, metformin can both directly and indirectly activate several core autophagy-related proteins, and in this way promote autophagy^15^. We revealed that treatment of fish with a low concentration of metformin (i.e., 50 µM) markedly induced the autophagic flux, especially at the early stage after cryoinjury (Fig. 2). Most strikingly, treatment with metformin accelerated epicardial regeneration (Fig. 4), endocardial regeneration, and revascularization (Fig. 5), as well as the deposition of collagen (Fig. 6), and stimulated the proliferation of cardiomyocytes and immature cardiomyocytes in the injured area (Fig. 3 and Supplementary Fig. 3). Moreover, this drug also transiently enhanced the ventricular systolic function as indicated by FS and EF, to increase the amount of blood being pumped out of the ventricle following injury (Supplementary Fig. 2). In contrast, when the autophagic flux was inhibited with CQ, then there was a concomitant delay in the regeneration of the epicardium, the endocardium, and vascular endothelium, as well as in the deposition of collagen along with the subsequent proliferation of cardiomyocytes and immature cardiomyocytes (Figs. 3–6). These results suggest that chronic metformin treatment can accelerate heart regeneration in adult zebrafish through the induction of autophagy. In humans, metformin has been shown to be safe and efficacious for the treatment of T2D, even when combined with other oral antidiabetic agents and insulin; thus when it is used at therapeutic doses, the side effects are rare^14^. Our results in fish, therefore, suggest that metformin might also have clinical value for the prevention and amelioration of MI in humans by enhancing autophagic activity in the heart.

mTOR is a serine/threonine-protein kinase in the phosphoinositide 3-kinase (PI3K)-related kinase (PIKK) signaling pathway, and it contains two functionally distinct complexes, mTORC1 and mTORC2, which coordinate cell growth, proliferation, and survival^58^. mTORC1 can phosphorylate ULK1 at residue Ser 757^17^ and TFEB at residue Ser 211^23,24^, thereby rendering them inactive. In contrast, the inhibition of mTORC1 activates ULK1^17^ and promotes TFEB nuclear translocation for lysosomal biogenesis^23,24^, which leads to enhanced autophagy. It has previously been suggested that inhibition of the mTOR pathway might be a therapeutic strategy for cardiomyopathy^59^. However, a more recent study showed that although treatment with rapamycin, an mTORC1 inhibitor, induced autophagy, it also impaired zebrafish heart regeneration after ventricular apex resection^37^. Interestingly, we showed that Torin 1 (another mTOR inhibitor) increased autophagy and it also accelerated regeneration of the zebrafish heart after cryoinjury (Supplementary Fig. 1). It is unclear why two different mTOR inhibitors generated contradictory results in two different heart regeneration fish models.

In the vertebrate species, fibrin, fibronectin, and hyaluronic acid initiate a temporary matrix after wounding, and then approximately 3 days post-wounding collagen III replaces the hyaluronic acid^60,61^. Concomitantly, proteins such as collagen IV and laminin in the basement membrane are synthesized during the early stages of wound healing^62^. These ECM components form a granulation tissue that is rich in heparin sulfate proteoglycans and chondroitin/dermatan proteoglycans; this tissue facilitates the replacement of collagen III by collagen I and it eventually matures into a fibrotic scar^63^. In humans, cardiac fibrosis is a significant global health problem associated with heart failure and other cardiovascular pathologies, such as MI^64^. However, in zebrafish, transient fibrosis is indispensable for heart regeneration^65^, and a deficiency of collagen due to the inhibition of TGFβ signaling impairs the regeneration of the heart^51^. Furthermore, the genetic ablation of collagen *1a2*-expressing cells influences cardiomyocyte proliferation in the context of zebrafish heart regeneration^52^. In our experiments, after heart cryoinjury, metformin treatment accelerated collagen deposition and heart regeneration, whereas CQ treatment delayed the deposition of collagen in the injured area and thus blocked heart regeneration (Fig. 6). Fibroblasts are responsible for homeostasis of the ECM, and thus they play a critical role in inflammation, the proliferation of non-myocytes, and scar maturation^53^. After an acute myocardial injury, diverse pro-inflammatory cytokines and pro-fibrotic factors are upregulated in fibroblasts, and these further promote the proliferation of fibroblasts. During this maturation phase, fibroblasts begin to secrete and accumulate collagens and other ECM proteins^66^. We showed that following heart cryoinjury, metformin treatment led to an increase in both the number of vimentin-positive fibroblasts and the autophagic flux in fibroblasts at 4 dpc, which resulted in more collagen deposition. In contrast, although CQ treatment still resulted in an accumulation of vimentin-positive fibroblasts in the injured area, autophagy was blocked in fibroblasts at 4 dpc and 7/ dpc (Fig. 6). We suggest that the increased number of fibroblasts might partially compensate for their aberrant production of collagen. In summary, the activation of autophagy in fibroblasts is necessary for transient fibrosis during zebrafish heart regeneration.

Following zebrafish heart cryoinjury, a tremendous quantity of cells are damaged in the injured area (Fig. 2 and Supplementary Fig. 4), and the immune system response is triggered, shortly recruiting inflammatory cells such as neutrophils and macrophages to the injured area for clearance of tissue debris; and neutrophils and macrophages also have been shown to be required for neovascularization, collagen deposition and resolution, and cardiomyocyte proliferation^67–72^. More detailed analyses revealed that neutrophils and macrophages are recruited from a very early stage, and neutrophils are far more abundant than macrophages^68,69^. Moreover, *tnfα*^+^ macrophages stimulate collagen deposition, whereas *tnfα*^-^ macrophages facilitate collagen resolution during zebrafish heart regeneration^71^. Furthermore, recent studies showed that macrophages express collagen and collagen-associated genes, directly contributing to collagen deposition^72^. Additionally, Wt1b affects the migration behavior of macrophages in the injured area, and *wt1b*^+^ macrophages are necessary for heart regeneration^70^. Accumulating evidence have suggested that metformin has a direct anti-inflammatory action. Through the activation of AMPK and inhibition of mTOR pathways, metformin suppresses inflammatory response by inhibition of the NFκB and PARP-1 pathways, as well as improvement of nitric oxide production and inhibition of advanced glycation endproducts (AGEs) formation and receptor for AGE expression; additionally, metformin regulates mitochondrial homeostasis by the elimination of defective mitochondria (mitophagy), which prevents the release of mitochondrial reactive oxygen species and mitochondrial DNA, resulting in the reduction of pro-inflammatory cytokines^73–75^. Metformin’s potential effect on inflammation in zebrafish heart regeneration is of interest because of the importance of these mechanisms in modulation of cellular health.

In summary, our results demonstrated that treatment with metformin accelerated zebrafish heart regeneration through the induction of autophagy, and there was a concomitant acceleration in the regeneration of the epicardium, the endocardium and vascular endothelium, and the myocardium, as well as in the deposition of collagen. These findings suggest that this first-line diabetic drug also has clinical value for the prevention and amelioration of MI in humans by enhancement of autophagy in the heart.

## Methods

### Zebrafish strains and maintenance

Wild type AB, *Tg*(*cmlc2*: *EGFP*), and *Tg*(*fli1a*: *EGFP*) lines of zebrafish were all acquired from the Zebrafish International Resource Center (ZIRC; University of Oregon, Eugene, OR, USA). The *Tg*(*cmv*:*GFP-LC3*) fish line was a kind gift from Prof. Jeff Bronstein (University of California, Los Angeles), and the *Tg*(*tcf21:DsRed2*) line was a kind gift from Prof. Jingwei Xiong (Peking University). The *ulk1b* mutant line was generated by TALEN, as described by Hasan et al^38^. Zebrafish were raised and maintained in 50 L glass tanks at a density of ~ 60 fish per tank. The water temperature was 28 ± 1 °C and the fish were kept on a constant 14 h light:10 h dark photoperiod daily. The water was changed twice a week, and the fish were fed with powdered food twice a day, and with brine shrimp once a day. All the procedures used in this study with live fish were performed in accordance with the guidelines and regulations set out by the Animal Research Ethics Committee of City University of Hong Kong and the Department of Health, Hong Kong.

### Cryoinjury of the zebrafish heart

Zebrafish aged 6–15 months were anesthetized in water containing 0.05% ethyl 3-aminobenzoate methanesulfonate (MS-222 or tricaine; E10521, Sigma-Aldrich). The ventricle was cryoinjured using a liquid nitrogen re-cooled probe, as described by Xu et al^76^. In a sham control group, the same protocol was followed but the heart was not subjected to cryoinjury.

### Pharmacological treatment

Stock solutions of 50 mM metformin (Alx-270-432; Enzo Life Sciences) and 100 mM chloroquine (C6628; Sigma-Aldrich) were prepared in distilled water, and stock solution of 25 µM Torin 1 (475991; EMD Chemical) was prepared in DMSO (D5879; Sigma-Aldrich). Just prior to use, the metformin, CQ, and Torin 1 stock solutions were diluted in fish water to final concentrations of 50 µM, 100 µM, and 25 nM, respectively.

### Protein extraction and western blotting

Zebrafish hearts at different time points (*n* = 3 per time point) were added to 200 µl ice-cold RIPA buffer (89900; Thermo Fisher Scientific) containing 1 mM PMSF (93482; Sigma-Aldrich). The concentration of extracted protein was determined using the BCA protein assay (23227; Thermo Fisher Scientific) according to the manufacturer’s instructions. For each sample, 20 µg protein was loaded onto a 15% or 8% SDS-polyacrylamide gel, after which the gel electrophoresis was performed using a Mini-Vertical Electrophoresis Cell system (1658005; Bio-Rad Laboratories); the system was run for 3 h under a constant voltage of 80 V. The separated proteins were then transferred from the gel to a PVDF membrane (10600023; GE Healthcare life science) using a Mini-Trans Blot Electrophoretic Transfer Cell system (1703930; Bio-Rad Laboratories) for 2 h under a constant voltage of 100 V. The PVDF membrane was then incubated in blocking buffer (PBST containing 5% non-fat milk powder) for 1 h at room temperature, followed by incubation with primary antibody overnight at 4 °C with constant shaking. The following primary antibodies were used: rabbit anti-LC3B (QH2069687; Thermo Fisher Scientific) diluted 1:2,000; mouse anti-GAPDH (60004-1; Proteintech Group) diluted 1:5,000; and rabbit anti-p-mTOR (2971 s; Cell Signaling Technology) diluted 1:2,000. The membrane was then washed with PBST and incubated with HRP-conjugated secondary antibody for 1 h at room temperature. The following secondary antibodies were used: goat anti-rabbit HRP (AP307P; Millipore Corp.) and rabbit anti-mouse HRP (AP160P; Millipore Corp.), both diluted 1:5,000. The protein of interest was detected with the EMD Millipore Luminata Western HRP chemiluminescence substrate (WBLUF0500; Millipore) and the Western blotting system (C600; Azure Biosystems), according to the manufacturer’s instructions. All western blots derive from the same experiment and that were processed in parallel.

### Histological techniques

Zebrafish hearts were harvested and fixed with PBS containing 4% PFA (158127; Sigma-Aldrich) for 3 h at room temperature. The fixed and dehydrated hearts were embedded in paraffin, after which sections of ~ 5 μm thickness were prepared. Serial sections were stained using a Picro Sirius Red stain kit (ab150681, Abcam) to label collagen. For each heart sample, the injured volume percentage in the ventricle was calculated according to the method described by Xu et al^69^. Some of the sections were stained with MSB to label fibrin and collagen. These sections were incubated with Martius yellow (287814; Sigma-Aldrich) for ~ 3 min and then rinsed with distilled water to remove the excess reagent. The sections were then incubated with crystal scarlet (96365; Sigma-Aldrich) for ~ 10 min, and then with phosphotungstic acid (P4006; Sigma-Aldrich) until the fibrin component turned red (this took ~ 15 min). The sections were then rinsed with distilled water again and incubated with methyl blue (M6900; Sigma-Aldrich) for ~ 20 min until the collagen component had turned blue. The sections were then rinsed briefly with 1% aqueous acetic acid, after which they were dehydrated rapidly, and mounted under coverslips with Histofluid mounting medium (6900002; Paul Marienfeld). Bright-field images were acquired using an Olympus DP72 digital camera mounted on an Olympus BX61 microscope.

In another series of experiments, sections containing injured heart areas were immunolabeled. The sections were dewaxed and rehydrated, and then antigen retrieval was performed by incubating them in sodium citrate buffer (10 mM sodium citrate, 0.05 % Tween 20, pH 6.0) at 95 °C for 20 min, after which they were cooled to room temperature. The sections were then incubated in blocking buffer (PBST containing 5% BSA) for 1 h, after which they were incubated with the appropriate primary antibody at 4 °C overnight. The following primary antibodies were used: rabbit anti-GFP (1891900; Life Technologies) at 1:200 dilution; mouse anti-PCNA (sc-7907; Santa Cruz Biotechnology) at 1:200 dilution; mouse anti-MF20 (Developmental Studies Hybridoma Bank; DSHB) at 1:50 dilution; mouse anti-embCMHC (N2.261; DSHB) at 1:50 dilution and mouse anti-vimentin (ab8978; Abcam) at 1:200 dilution. After incubation with the primary antibody, the sections were washed with PBST and then incubated with the appropriate secondary antibody for 2 h at room temperature. The following secondary antibodies were used: Goat anti-rabbit FITC (F6005; Sigma-Aldrich) at 1:200 dilution, and goat anti-mouse Cy3 (1855013; Life Technologies) at 1:200 dilution. The sections were then incubated with DAPI (10236276001, Roche Diagnostics) at 1:10,000 dilution in PBST for 10 min, and then washed with PBST. The sections were mounted under coverslips with antifade mountant (P36934; Life Technologies), and fluorescence images were acquired using a Hamamatsu ORCA-Flash4.0 digital camera mounted on the Olympus BX61 microscope. To visualize apoptosis, some sections were labeled with a TUNEL assay kit (G3250; Promega), according to the manufacturer’s instructions.

### Transmission electron microscopy

Zebrafish hearts were dissected and fixed with a freshly prepared primary fixative containing 2.5% glutaraldehyde (16020; Electron Microscopy Sciences) and 2% PFA for 2 to 3 h at room temperature. The fixed hearts were then rinsed with PBS, after which they were fixed with a freshly prepared secondary fixative containing 2% osmium tetroxide (19170; Electron Microscopy Sciences) in the dark for 2 h at room temperature. The fixed and dehydrated hearts were then embedded in Spurr’s resin (14300; Electron Microscopy Sciences). Ultra-thin sections (~ 70 nm thickness) were cut with an ultramicrotome (Ultracut UCT; Leica Biosystems) and collected on formvar-coated grids. They were then stained with lead citrate (17800; Electron Microscopy Sciences) and uranyl acetate (22400; Electron Microscopy Sciences). TEM images were acquired with a Philips Tecnai 12 BioTWIN transmission electron microscope (FEI Company).

### Light sheet fluorescence microscopy

The *Tg*(*tcf21:DsRed2*) line of fish was used in these experiments, where DsRed2 fluorescence is expressed specifically in the epicardium. Zebrafish were sacrificed and the hearts were harvested at 1 dpc, 4 dpc, 7 dpc, 14 dpc, and 21 dpc. Each heart was mounted in a glass capillary with an inner diameter of ~ 1.5 mm (507068; Brand GmbH) using 1% low melting point agarose (50080; Lonza Rockland). The capillary was assembled into a sample holder, and this was then inserted into a sample chamber filled with PBS at room temperature. A Z-stack series of frontal view optical sections of the ventricular apex were then acquired using a light sheet fluorescence microscope (Lightsheet Z.1; Carl Zeiss Microscopy) and a 5 × water objective lens. The Z-stack series of images were then displayed in three dimensions using the surface render mode, according to the manufacturer’s instructions.

### Echocardiography

The hearts of wild-type AB strain zebrafish aged 15 months were cryoinjured, and then 6 fish (3 female and 3 male) were maintained in untreated fish water or in fish water containing 50 µM metformin. Each fish was anesthetized in water containing 0.01 % tricaine for ~ 3 min, after which it was placed ventral side up in a 0.5 cm × 5 cm groove cut in the middle of a piece of damp sponge in a beaker. Echocardiography was acquired using a Vevo LAZR Multi-modality Imaging Platform (FUJIFILM VisualSonics) in B-mode (50 MHz, 77 fps), with an ultrasound transducer fixed above the ventral side of the zebrafish and parallel to the longitudinal axis (LAX) plane. Color Doppler (40 MHz, 25 kHz PRF) was used to precisely measure the direction of blood inflow from the atrium. The whole imaging procedure took no more than 10 min. The fish was then returned to fresh fish water where it could recover from the anesthetic. Each fish was maintained in fish water until the next time point when echocardiography was conducted again. B-mode images were analyzed using a Vevo LAB (Vevo Strain) plug-in, and the window was set to be 300 frames long, starting with the end-diastole. Ventricular chamber dimensions were obtained from B-mode images in the LAX view, and systolic function was measured as fractional shortening and ejection fraction.

### Statistical analysis

The results were generated from at least 3 independent experiments. The injured area and fluorescent signals in the injured area were quantified with ImageJ. All data assumptions of normality were tested. We did not observe any deviation from the normality of variance that would justify the use of non-parametric tests. Data were expressed as mean ± standard deviation. To compare differences between the experimental and control groups, the independent samples T-test or one-way ANOVA with LSD *post hoc* was used and data with *p* < 0.05 were considered to be significant. All statistical analyses were performed using IBM SPSS Statistics 20.

### Reporting Summary

Further information on research design is available in the Nature Research Reporting Summary linked to this article.

## Supplementary information

Supplementary Information

Supplementary Video 1

Supplementary Video 2

Supplementary Video 3

Supplementary Video 4

Supplementary Video 5

Supplementary Video 6

Supplementary Video 7

Supplementary Video 8

Supplementary Video 9

Supplementary Video 10

Reporting Summary

## Data Availability

All data supporting the findings of this study are available from the corresponding authors upon reasonable request.
