# Supplementary material for: Metformin accelerates zebrafish heart regeneration by inducing autophagy
Source: NPJ Regen Med. 2021 Oct 8;6:62. doi: 10.1038/s41536-021-00172-w (PMC8501080; doi:10.1038/s41536-021-00172-w)
Supplement: Supplementary file 1 — Supplementary Information [file 41536_2021_172_MOESM1_ESM.pdf]

Supplementary Figures

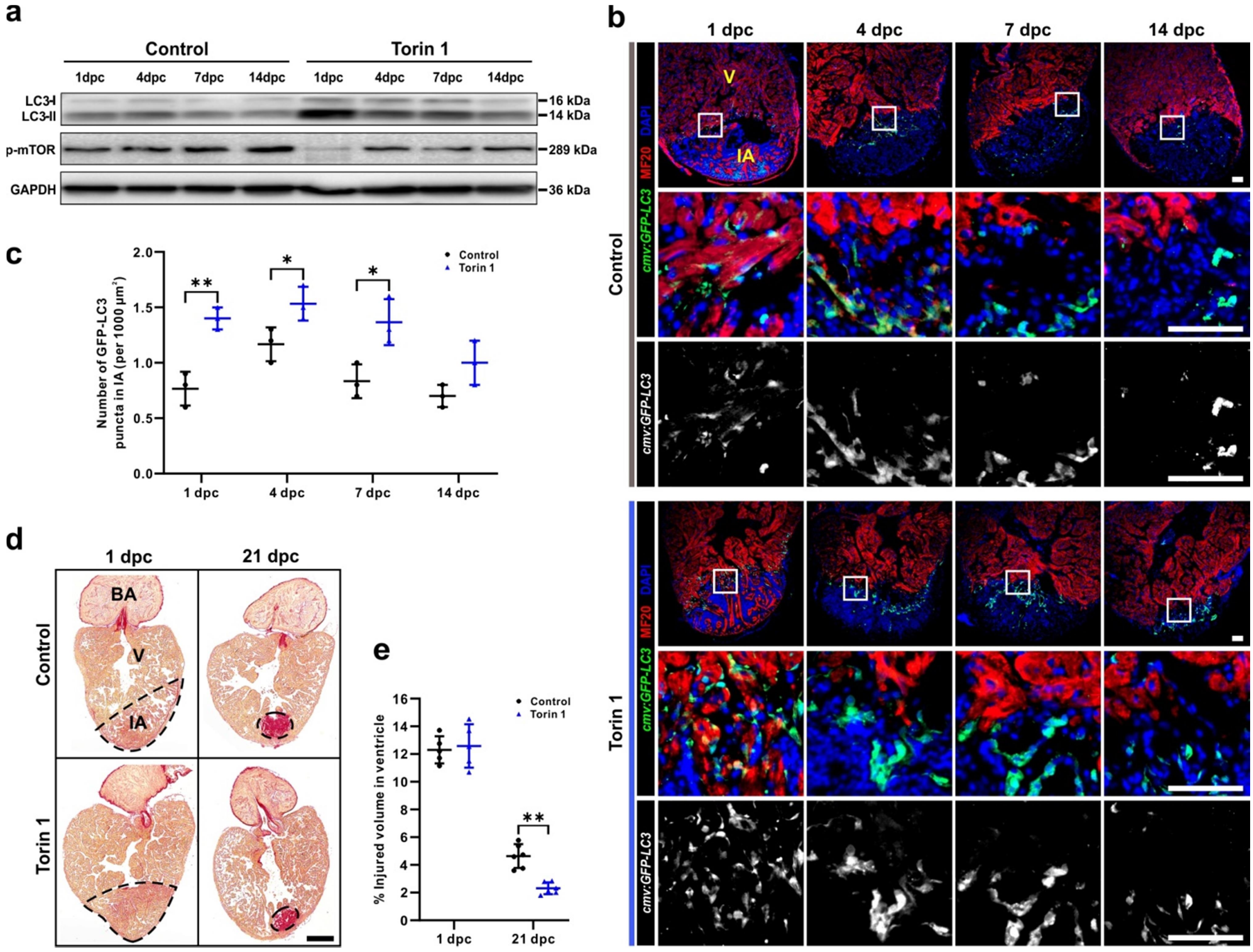

Supplementary Figure 1. Torin 1 enhances autophagic flux and accelerates heart regeneration after cryoinjury. (**a**) A western blot analysis was performed to show the expression of LC3-I, LC3-II and p-mTOR from 1 dpc to 14 dpc following treatment with 25 nM Torin 1 when compared with a DMSO control. The protein from 3 hearts was loaded into each lane, and GAPDH was used as the loading control. (**b, c**) In this experiment, the *Tg*(*cmv*:*GFP-LC3*) line of fish was used. Sectioned hearts were dual-immunostained with an anti-GFP antibody to label LC3 (in green) and an anti-MF20 antibody to label the cardiomyocytes (in red), and then the nuclei were labeled with DAPI (in blue). V: ventricle; IA: injured area. Scale bars: 50 μm (**b**). The number of GFP-LC3 puncta in the heart of control and Torin 1-treated fish at 1 dpc, 4 dpc, 7 dpc and 14 dpc was quantified. The data are presented as mean ± SD, n = 3 hearts, $*P < 0.05$, $**P < 0.01$ *vs* control (**c**). (**d, e**) AB fish were either untreated (control) or else treated with 25 nM Torin 1 for up to 21 days following cryoinjury after which the hearts were isolated, and paraffin sections prepared and stained with picro sirius red. BA: bulbous arteriosus. Scale bar: 200 μm (**d**). The injured volume percentage in the ventricle was quantified for the control and Torin 1-treatment groups at 1 dpc and 21 dpc. The data are expressed as mean ± SD, n = 5 to 6 hearts, $**P < 0.01$ *vs* control (**e**).

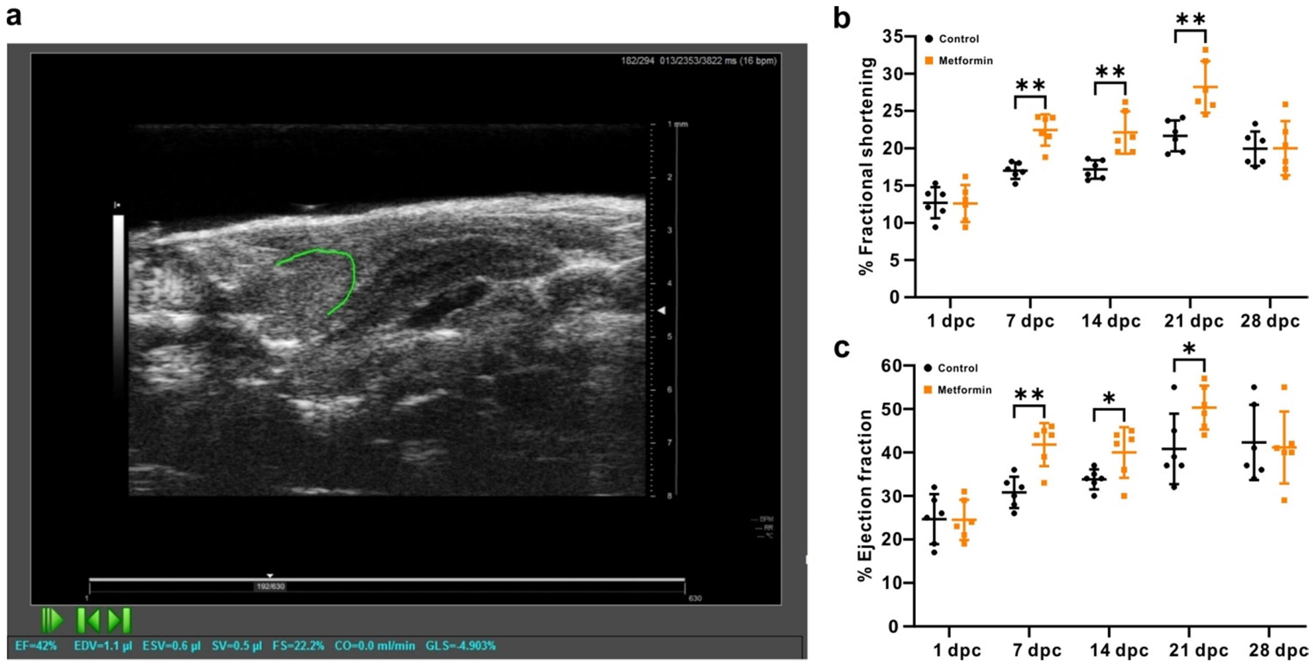

Supplementary Figure 2. Metformin treatment promotes systolic function during heart regeneration. (**a**) A representative longitudinal ventricular assessment of wild type AB fish by B-Mode imaging. The hearts were cryoinjured, and the fish were maintained in untreated fish water (control) or in fish water containing 50 µM metformin, and then cardiac function was acquired at 1 dpc, 7 dpc, 14 dpc, 21 dpc and 28 dpc, using high resolution, real-time echocardiography. The inner border of the compact myocardium is delineated by the green line. (**b, c**) Bar graphs showing the fractional shortening (FS) (**b**) and ejection fraction (EF) (**c**) measured in the control and metformin treated fish. The data are expressed as mean ± SD, n = 6 hearts (3 female and 3 male), *$P < 0.05$, **$P < 0.01$ *vs* control.

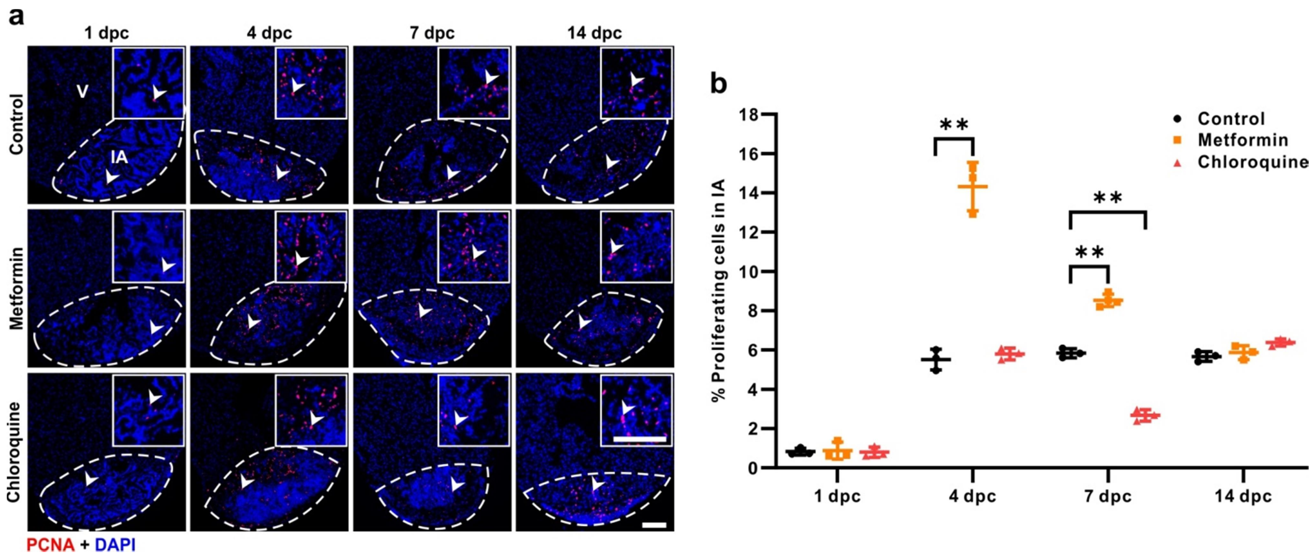

Supplementary Figure 3. Metformin increases the amount of cell proliferation after cryoinjury. (**a**) After cryoinjury, wild type AB fish were untreated (control) or were treated with 50 µM metformin or 100 µM CQ. The hearts were isolated at 1 dpc, 4 dpc, 7 dpc and 14 dpc, and sections were prepared and immunostained with an anti-PCNA antibody for the identification of proliferating cells (in red) and then co-labeled with DAPI to label the nuclei (in blue). V: ventricle; IA: injured area. The panels in the upper right corner of each image show higher magnification views of regions of the IA with PCNA-positive cells. Scale bars: 100 µm. (**b**) Bar graph showing the percentage of proliferating cells in the injured area in the untreated control, metformin, or CQ treated fish at 1 dpc, 4 dpc, 7 dpc and 14 dpc. The data are expressed as mean ± SD, n = 3 to 4 hearts, $^{**}P < 0.01$ *vs* control.

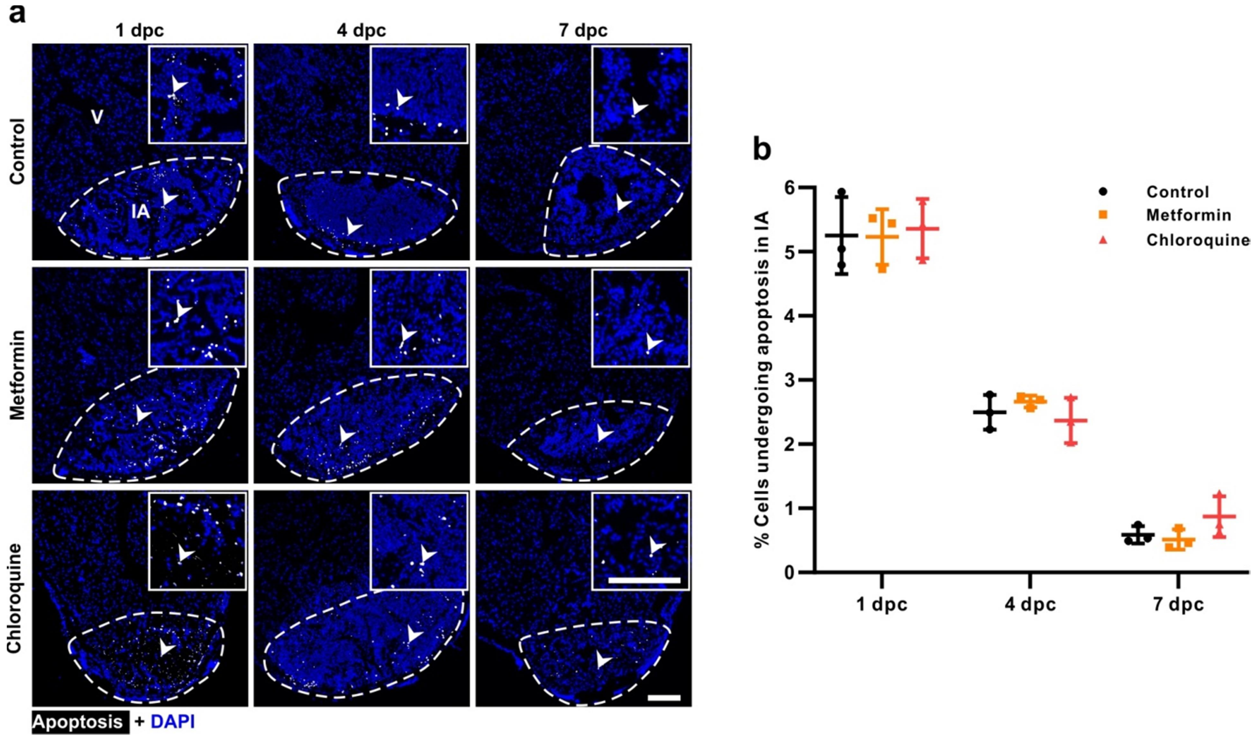

Supplementary Figure 4. The number of apoptotic cells in the injured area is similar in control and metformin- or chloroquine-treated fish. (**a**) After cryoinjury, wild type AB fish were untreated (control) or were treated with 50 µM metformin or 100 µM chloroquine. The hearts were isolated at 1 dpc, 4 dpc, and 7 dpc, and sections were prepared and labeled with the components of the TUNEL assay to identify apoptotic cells (in white), and then co-labeled with DAPI to show the nuclei (in blue). V: ventricle; IA: injured area. Scale bars: 100 µm. (**b**) Bar graph showing the percentage of apoptotic cells in the IA in the control and metformin- or CQ-treated fish at 1 dpc, 4 dpc and 7 dpc. The data are expressed as mean ± SD, n = 3 hearts. The number of apoptotic cells decreased from 1 dpc to 7 dpc, but no significant differences were observed.

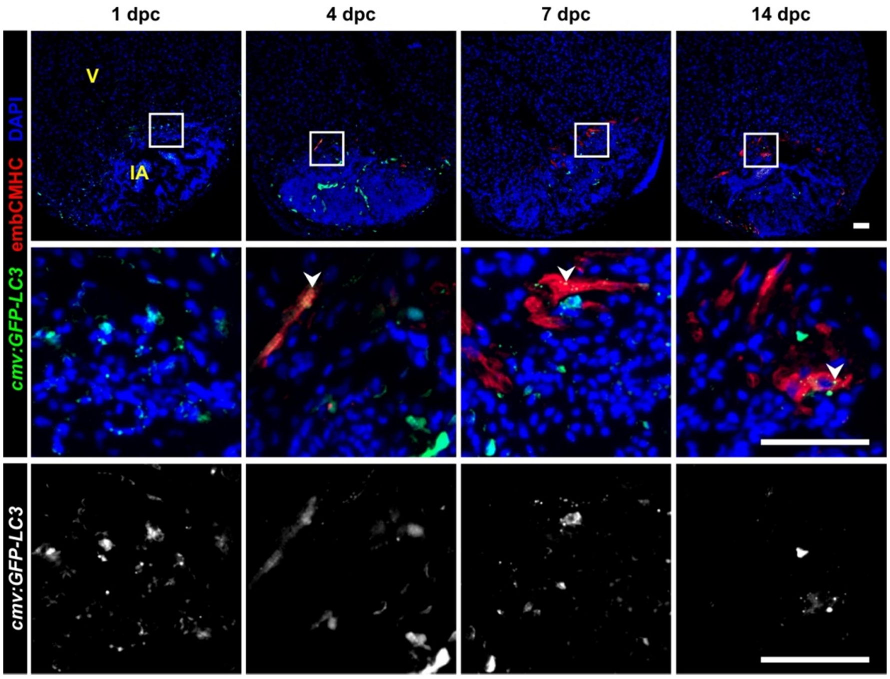

Supplementary Figure 5. Autophagy is activated in embryonic cardiomyocytes. After cryoinjury, the hearts from *Tg*(*cmv*:*GFP-LC3*) line of fish were isolated at 1 dpc, 4 dpc, 7 dpc and 14 dpc, and sections were prepared and dual-immunostained with an anti-GFP antibody to mark LC3 (in green) and an anti-embCMHC (N2.261) antibody for the identification of embryonic cardiac myosin heavy chain (in red), after which the nuclei were labeled with DAPI (in blue). V: ventricle; IA: injured area. Scale bars: 50 μm. GFP-LC3 puncta were detected in the embryonic cardiomyocytes (see white arrowheads) at 4 dpc, 7 pdc and 14 dpc.

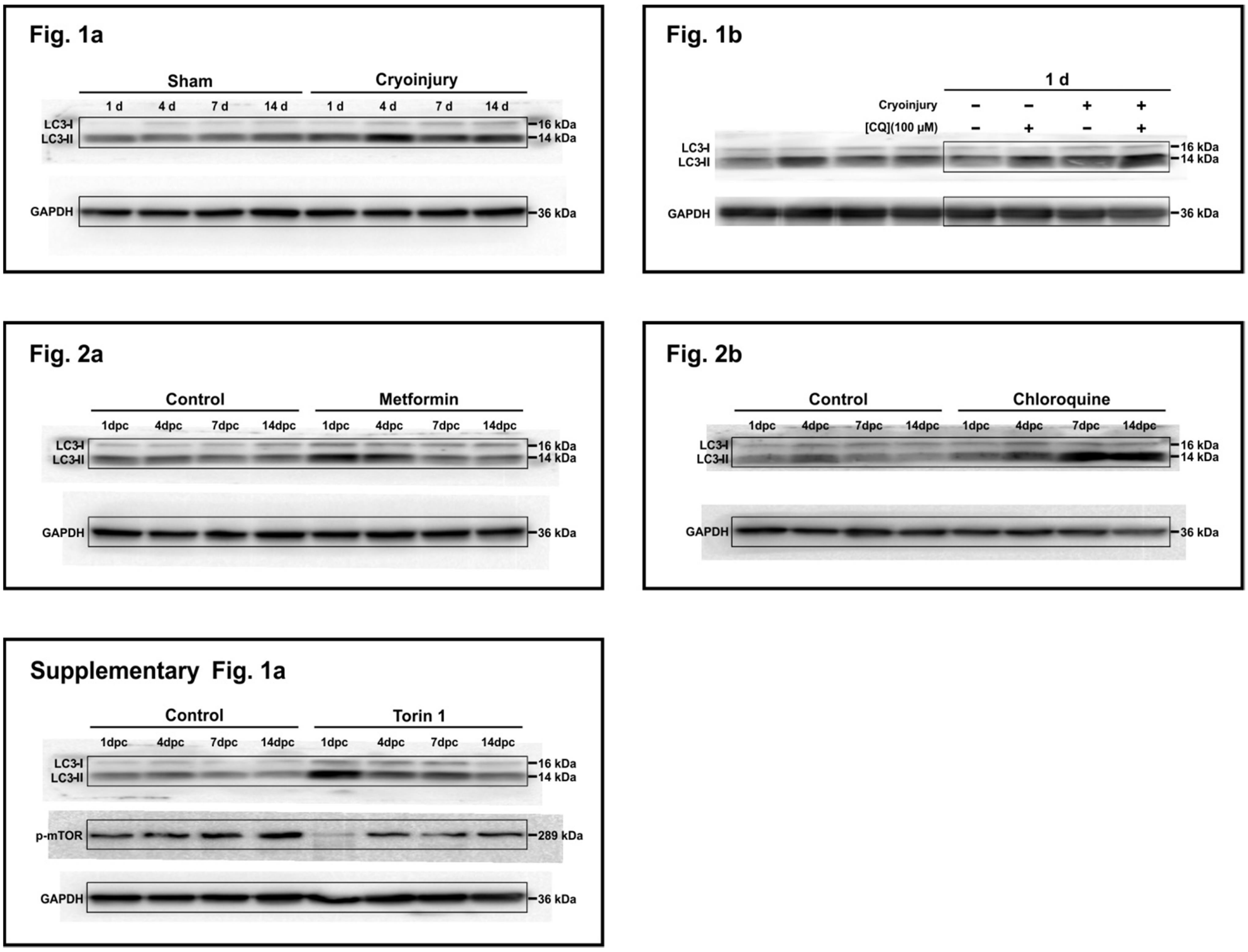

Supplementary Figure 6. Un-cropped images of western blots relate to Figure 1, Figure 2 and Supplementary Figure 1.

Supplementary Video Legends

Supplementary Video 1. Longitudinal axis B-mode video of an untreated zebrafish at 1 dpc. The hearts of wild type AB strain zebrafish aged 15 months were cryoinjured, and the fish were maintained in untreated fish water (control). At 1 dpc, echocardiography was acquired using a Vevo LAZR Multi-modality Imaging Platform (FUJIFILM VisualSonics) in B-mode (50 MHz, 77 fps), with an ultrasound transducer fixed above the ventral side of the zebrafish and parallel to the longitudinal axis plane.

Supplementary Video 2. Longitudinal axis B-mode video of a metformin-treated zebrafish at 1 dpc. The hearts of wild type AB strain zebrafish aged 15 months were cryoinjured, and the fish were maintained in fish water containing 50 μM metformin. At 1 dpc, echocardiography was acquired using a Vevo LAZR Multi-modality Imaging Platform (FUJIFILM VisualSonics) in B-mode (50 MHz, 77 fps), with an ultrasound transducer fixed above the ventral side of the zebrafish and parallel to the longitudinal axis plane.

Supplementary Video 3. Longitudinal axis B-mode video of an untreated zebrafish at 7 dpc. The hearts of wild type AB strain zebrafish aged 15 months were cryoinjured, and the fish were maintained in untreated fish water (control). At 7 dpc, echocardiography was acquired using a Vevo LAZR Multi-modality Imaging Platform (FUJIFILM VisualSonics) in B-mode (50 MHz, 77 fps), with an ultrasound transducer fixed above the ventral side of the zebrafish and parallel to the longitudinal axis plane.

Supplementary Video 4. Longitudinal axis B-mode video of a metformin-treated zebrafish at 7 dpc. The hearts of wild type AB strain zebrafish aged 15 months were cryoinjured, and the fish were maintained in fish water containing 50 μM metformin. At 7 dpc, echocardiography was acquired using a Vevo LAZR Multi-modality Imaging Platform (FUJIFILM VisualSonics) in B-mode (50 MHz, 77 fps), with an ultrasound transducer fixed above the ventral side of the zebrafish and parallel to the longitudinal axis plane.

Supplementary Video 5. Longitudinal axis B-mode video of an untreated zebrafish at 14 dpc. The hearts of wild type AB strain zebrafish aged 15 months were cryoinjured, and the fish were maintained in untreated fish water (control). At 14 dpc, echocardiography was acquired using a Vevo LAZR Multi-modality Imaging Platform (FUJIFILM VisualSonics) in B-mode (50 MHz, 77 fps), with an ultrasound transducer fixed above the ventral side of the zebrafish and parallel to the longitudinal axis plane.

Supplementary Video 6. Longitudinal axis B-mode video of a metformin-treated zebrafish at 14 dpc. The hearts of wild type AB strain zebrafish aged 15 months were cryoinjured, and the fish were maintained in fish water containing 50 μM metformin. At 14 dpc, echocardiography was acquired using a Vevo LAZR Multi-modality Imaging Platform (FUJIFILM VisualSonics) in B-mode (50 MHz, 77 fps), with an ultrasound transducer fixed above the ventral side of the zebrafish and parallel to the longitudinal axis plane.

Supplementary Video 7. Longitudinal axis B-mode video of an untreated zebrafish at 21 dpc. The hearts of wild type AB strain zebrafish aged 15 months were cryoinjured, and the fish were maintained in untreated fish water (control). At 21 dpc, echocardiography was acquired using a Vevo LAZR Multi-modality Imaging Platform (FUJIFILM VisualSonics) in B-mode (50 MHz, 77 fps), with an ultrasound transducer fixed above the ventral side of the zebrafish and parallel to the longitudinal axis plane.

Supplementary Video 8. Longitudinal axis B-mode video of a metformin-treated zebrafish at 21 dpc. The hearts of wild type AB strain zebrafish aged 15 months were cryoinjured, and the fish were maintained in fish water containing 50 μM metformin. At 21 dpc, echocardiography was acquired using a Vevo LAZR Multi-modality Imaging Platform (FUJIFILM VisualSonics) in B-mode (50 MHz, 77 fps), with an ultrasound transducer fixed above the ventral side of the zebrafish and parallel to the longitudinal axis plane.

Supplementary Video 9. Longitudinal axis B-mode video of an untreated zebrafish at 28 dpc. The hearts of wild type AB strain zebrafish aged 15 months were cryoinjured, and the fish were maintained in untreated fish water (control). At 28 dpc, echocardiography was acquired using a Vevo LAZR Multi-modality Imaging Platform (FUJIFILM VisualSonics) in B-mode (50 MHz, 77 fps), with an ultrasound transducer fixed above the ventral side of the zebrafish and parallel to the longitudinal axis plane.

Supplementary Video 10. Longitudinal axis B-mode video of a metformin-treated zebrafish at 28 dpc. The hearts of wild type AB strain zebrafish aged 15 months were cryoinjured, and the fish were maintained in fish water containing 50 μM metformin. At 28 dpc, echocardiography was acquired using a Vevo LAZR Multi-modality Imaging Platform (FUJIFILM VisualSonics) in B-mode (50 MHz, 77 fps), with an ultrasound transducer fixed above the ventral side of the zebrafish and parallel to the longitudinal axis plane.
