# Supplementary material for: Metformin accelerates zebrafish heart regeneration by inducing autophagy
Source: NPJ Regen Med. 2021 Oct 8;6:62. doi: 10.1038/s41536-021-00172-w (PMC8501080; doi:10.1038/s41536-021-00172-w)
Supplement: Supplementary file 12 — Reporting Summary [file 41536_2021_172_MOESM12_ESM.pdf]

nature portfolio

Corresponding author(s): Shuk Han Cheng
Last updated by author(s): Aug 18, 2021

# Reporting Summary

Nature Portfolio wishes to improve the reproducibility of the work that we publish. This form provides structure for consistency and transparency in reporting. For further information on Nature Portfolio policies, see our Editorial Policies and the Editorial Policy Checklist.

## Statistics

For all statistical analyses, confirm that the following items are present in the figure legend, table legend, main text, or Methods section.

| n/a | Confirmed | |
|---|---|---|
| ☐ | ☒ | The exact sample size ($n$) for each experimental group/condition, given as a discrete number and unit of measurement |
| ☐ | ☒ | A statement on whether measurements were taken from distinct samples or whether the same sample was measured repeatedly |
| ☐ | ☒ | The statistical test(s) used AND whether they are one- or two-sided<br>*Only common tests should be described solely by name; describe more complex techniques in the Methods section.* |
| ☒ | ☐ | A description of all covariates tested |
| ☐ | ☒ | A description of any assumptions or corrections, such as tests of normality and adjustment for multiple comparisons |
| ☐ | ☒ | A full description of the statistical parameters including central tendency (e.g. means) or other basic estimates (e.g. regression coefficient) AND variation (e.g. standard deviation) or associated estimates of uncertainty (e.g. confidence intervals) |
| ☐ | ☒ | For null hypothesis testing, the test statistic (e.g. $F$, $t$, $r$) with confidence intervals, effect sizes, degrees of freedom and $P$ value noted<br>*Give P values as exact values whenever suitable.* |
| ☒ | ☐ | For Bayesian analysis, information on the choice of priors and Markov chain Monte Carlo settings |
| ☒ | ☐ | For hierarchical and complex designs, identification of the appropriate level for tests and full reporting of outcomes |
| ☒ | ☐ | Estimates of effect sizes (e.g. Cohen's $d$, Pearson's $r$), indicating how they were calculated |

*Our web collection on statistics for biologists contains articles on many of the points above.*

## Software and code

Policy information about availability of computer code

| | |
|---|---|
| Data collection | No software was used. |
| Data analysis | No software was used. |

For manuscripts utilizing custom algorithms or software that are central to the research but not yet described in published literature, software must be made available to editors and reviewers. We strongly encourage code deposition in a community repository (e.g. GitHub). See the Nature Portfolio guidelines for submitting code & software for further information.

## Data

Policy information about availability of data

All manuscripts must include a data availability statement. This statement should provide the following information, where applicable:

- Accession codes, unique identifiers, or web links for publicly available datasets
- A description of any restrictions on data availability
- For clinical datasets or third party data, please ensure that the statement adheres to our policy

All data supporting the findings of this study are available from the corresponding authors upon reasonable request.

# Field-specific reporting

Please select the one below that is the best fit for your research. If you are not sure, read the appropriate sections before making your selection.

- [x] Life sciences
- [ ] Behavioural & social sciences
- [ ] Ecological, evolutionary & environmental sciences

For a reference copy of the document with all sections, see nature.com/documents/nr-reporting-summary-flat.pdf

# Life sciences study design

All studies must disclose on these points even when the disclosure is negative.

| | |
|---|---|
| Sample size | Sample sizes were determined based on our preliminary experiments. In our experience, each experiment were designed and repeat at least 3 times. For imaging, at least 3 zebrafish per group per time were used, which was sufficient to detect meaningful biological differences with good reproducibility. |
| Data exclusions | No data were exclused from the analyses. |
| Replication | All attempts at replication were successful. |
| Randomization | All samples were randomly allocated into experimental groups. |
| Blinding | We were blinded to group allocation during data analysis. |

# Reporting for specific materials, systems and methods

We require information from authors about some types of materials, experimental systems and methods used in many studies. Here, indicate whether each material, system or method listed is relevant to your study. If you are not sure if a list item applies to your research, read the appropriate section before selecting a response.

## Materials & experimental systems

| n/a | Involved in the study | |
|---|---|---|
| ☐ | ☒ | Antibodies |
| ☒ | ☐ | Eukaryotic cell lines |
| ☒ | ☐ | Palaeontology and archaeology |
| ☐ | ☒ | Animals and other organisms |
| ☒ | ☐ | Human research participants |
| ☒ | ☐ | Clinical data |
| ☒ | ☐ | Dual use research of concern |

## Methods

| n/a | Involved in the study | |
|---|---|---|
| ☒ | ☐ | ChIP-seq |
| ☒ | ☐ | Flow cytometry |
| ☒ | ☐ | MRI-based neuroimaging |

## Antibodies

| | |
|---|---|
| Antibodies used | The following primary antibodies were used: rabbit anti-LC3B (QH2069687; Thermo Fisher Scientific) diluted 1:2,000; mouse anti-GAPDH (60004-1; Proteintech Group) diluted 1:5,000; and rabbit anti-p-mTOR (2971s; Cell Signaling Technology) diluted 1:2,000； rabbit anti-GFP (1891900; Life Technologies) at 1:200 dilution; mouse anti-PCNA (sc-7907; Santa Cruz Biotechnology) at 1:200 dilution; mouse anti-MF20 (Developmental Studies Hybridoma Bank; DSHB) at 1:50 dilution; mouse anti-embCMHC (N2.261; DSHB) at 1:50 dilution and mouse anti-vimentin (ab8978; Abcam) at 1:200 dilution. The following secondary antibodies were used: goat anti-rabbit HRP (AP307P; Millipore Corp.) and rabbit anti-mouse HRP (AP160P; Millipore Corp.), both diluted 1:5,000； Goat anti-rabbit FITC (F6005; Sigma-Aldrich) at 1:200 dilution, and goat anti-mouse Cy3 (1855013; Life Technologies) at 1:200 dilution. |
| Validation | The validation of each primary antibody for zebrafish and application was provided in the manuscript. |

## Animals and other organisms

Policy information about studies involving animals; ARRIVE guidelines recommended for reporting animal research

| | |
|---|---|
| Laboratory animals | In this study, we used wild type AB, Tg(cmlc2: EGFP), Tg(fli1a: EGFP), Tg(cmv:GFP-LC3), Tg(tcf21:DsRed2) and the ulk1b mutant lines of zebrafish aged 6-15 months. |
| Wild animals | This study did not involve wild animals. |
| Field-collected samples | This study did not involve samples collected from the field. |

| | |
|---|---|
| Ethics oversight | All the procedures used in this study with live zebrafish were performed in accordance with the guidelines and regulations set out by the Animal Research Ethics Committee of City University of Kong Kong and the Department of Health, Hong Kong. |

Note that full information on the approval of the study protocol must also be provided in the manuscript.
